# Toposelective Functionalization of Solution‐Processed Transition Metal Dichalcogenides with Metal Nanoparticles via Defect Engineering

**DOI:** 10.1002/adma.202506605

**Published:** 2025-08-16

**Authors:** Stefano Ippolito, Verónica Montes‐García, Adam G. Kelly, Valentina Girelli Consolaro, Walid Baaziz, María José Cordero‐Ferradás, Arezoo Dianat, Jorge Pérez‐Juste, Isabel Pastoriza‐Santos, Ovidiu Ersen, Gianaurelio Cuniberti, Jonathan N. Coleman, Paolo Samorì

**Affiliations:** ^1^ ISIS UMR 7006 Université de Strasbourg & CNRS 8 allée Gaspard Monge Strasbourg F‐67000 France; ^2^ i3N/CENIMAT Faculty of Science and Technology Campus de Caparica Universidade NOVA de Lisboa Caparica 2829‐516 Portugal; ^3^ CNRS IPCMS Université de Strasbourg 23 rue du Loess BP 43 Cedex 2 Strasbourg 67034 France; ^4^ CINBIO Campus Universitario Lagoas‐Marcosende Universidade de Vigo Vigo 36310 Spain; ^5^ Institute for Materials Science and Max Bergmann Center of Biomaterials TU Dresden 01062 Dresden Germany; ^6^ School of Physics Centre for Research on Adaptive Nanostructures and Nanodevices (CRANN) and Advanced Materials and Bioengineering Research (AMBER) Trinity College Dublin Dublin 2 Ireland

**Keywords:** 2D materials, defect engineering, mixed‐dimensional materials, multifunctional hybrid system

## Abstract

Solution‐processed semiconducting transition metal dichalcogenides commonly serve as quintessential 2D substrates and templates to develop hybrid structures with novel and/or enhanced properties and performance. However, the effects and control of their ubiquitous and abundant structural defects are still poorly explored and understood. Here, exploiting their highly reactive and defective edges, an unprecedented strategy is introduced for their toposelective functionalization with noble metal nanoparticles through galvanic displacement. Selectively edge‐decorated transition metal dichalcogenides nanosheets are successfully produced with gold, palladium, or platinum nanoparticles, showing tunable loading and size. As proof of concept, the hybrid systems are tested for optical and photothermal sensing, as well as electrocatalysis and electronics, demonstrating their enhanced functionality and broad applicability. These findings pave the way for the versatile production of mixed‐dimensional multifunctional materials, achieved by harnessing the defective nature of solution‐processed transition metal dichalcogenides via molecular chemistry approaches.

## Graphical abstract

This work presents a novel strategy for toposelective functionalization of solution‐processed transition metal dichalcogenides with noble metal nanoparticles via galvanic displacement. Owing to the selective edge decoration with gold, palladium, or platinum nanoparticles, the hybrid materials exhibit new properties and improved device performance in sensing, electrocatalysis, and electronics. This approach exploits a defect engineering strategy to produce multifunctional mixed‐dimensional nanomaterials.

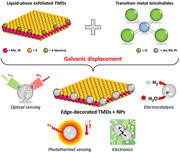

## 1 Introduction

Recent advances in the production of 2D nanosheets via solution processing have enlarged their range of applicability across various fields, spanning from electronics and catalysis to sensing and biomedicine.^[^ ^1^, ^2^, ^3^, ^4^ ^]^ Among them, semiconducting transition metal dichalcogenides (TMDs) have gained extensive attention owing to their wide‐ranging portfolio of physiochemical properties,^[^ ^5^ ^]^ which can be further expanded when they are used as building blocks to produce hybrid systems with novel and/or enhanced properties and performance.^[^ ^6^, ^7^, ^8^ ^]^ During the last decade, tremendous efforts have been devoted to exploiting the surface chemistry of TMDs through multiple approaches,^[^ ^9^, ^10^ ^]^ mostly relying on laborious phase engineering as well as complex and time‐consuming molecular functionalization strategies that often require harsh synthetic conditions.^[^ ^11^, ^12^ ^]^ More recently, an alternative chemical route has been consolidated, leveraging the specific interaction between thiolated molecules and the most abundant reactive sites in transition metal disulfides, namely sulfur vacancies (V_S_), that strongly impact the material's optoelectronic properties and, therefore, their device performance.^[^ ^13^ ^]^ In solution‐processed materials, V_S_ are primarily located at the flake edges and are produced during the exfoliation process. These reactive sites are present in high density and can be exploited during the subsequent functionalization protocols. The use of thiolated molecules has proven to be an effective and versatile strategy for the healing of V_S_ in TMDs, enhancing the performance of electronic devices by reducing the density of structural defects.^[^ ^14^, ^15^ ^]^ In solution‐processed materials, the performance of electrical devices is often hindered by poor inter‐flake electronic connectivity. This major limitation can be addressed using appropriately designed π‐conjugated dithiolated molecules, which simultaneously heal V_S_ and covalently bridge adjacent flakes.^[^ ^16^ ^]^ However, thiols are toxic chemicals and entail the use of strict operating conditions during the functionalization steps (e.g., inert atmosphere, dark conditions) and often necessitate extended reaction times. Therefore, it is highly desirable and needed to develop alternative molecular chemistry approaches, aiming at harnessing the reactive defect sites in TMDs for the production of hybrid multifunctional materials with enhanced properties and broad application potential.

**Figure 1:**
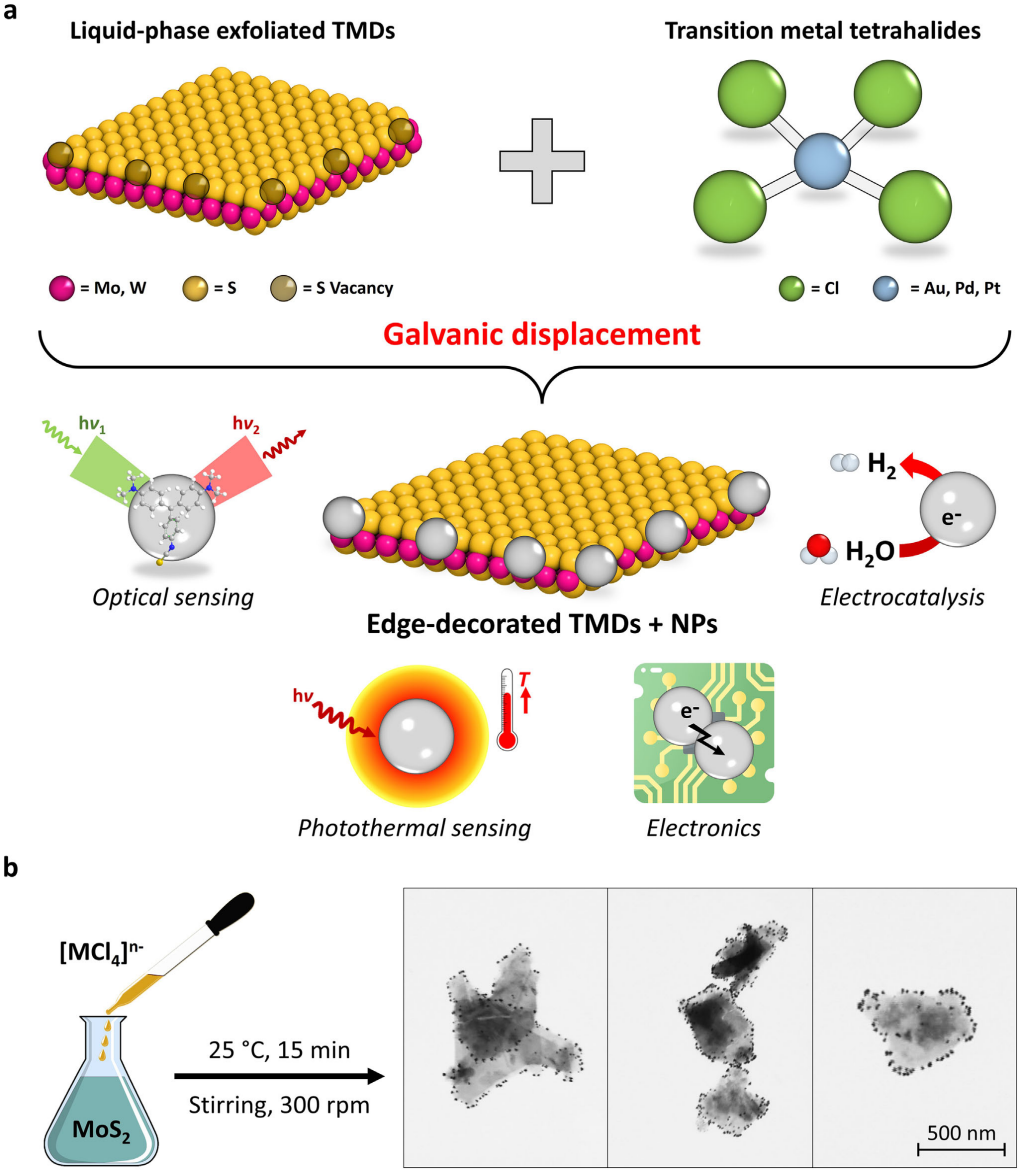
Toposelective functionalization of solution‐processed TMDs. a) Schematic of the GD reaction between solution‐processed TMDs and transition metal tetrahalides, leading to selectively edge‐decorated nanosheets with noble metal NPs. b) Illustration of the experimental conditions for the GD reaction (left), along with three scanning transmission electron microscopy (STEM) images (with the same magnification) showing MoS_2_ + AuNPs nanosheets (right).

Here, we report an innovative functionalization protocol for solution‐processed TMDs, leading to the formation of selectively edge‐decorated nanosheets with noble metal nanoparticles (NPs) through a defect‐engineered strategy based on galvanic displacement (GD). Such a versatile toposelective approach exploits the reaction between defects at the flake edges, mainly V_S_, and transition metal tetrachloride complexes of gold (Au), palladium (Pd), and platinum (Pt), namely HAuCl_4_, K_2_PdCl_4_, and K_2_PtCl_4_, respectively, acting as precursors for the growth of noble metal particles (**Figure** **1**a). Our method produces hybrid systems where the loading and size of the decorating NPs can be tailored *on demand*, simply by adjusting the stoichiometric ratio between the 2D material and coordination complex. Most importantly, owing to the main characteristic of GD (i.e., electroless deposition), no external reducing agent is needed, unlike conventional protocols, since the TMD nanosheets themselves play this primary role by providing the electrons needed to reduce the molecular metal precursors to metal NPs.^[^ ^17^ ^]^ Additionally, the absence of reducing or capping agents allows the formation of clean, uncapped NPs, which are highly accessible for catalytic, electronic, and sensing applications. Moreover, our strategy to obtain mixed‐dimensional hybrid structures (Figure 1b) is fast (viz., less than 15 min) and scalable, it entails only the use of aqueous solutions and it takes place at room temperature (RT), unlike more traditional methods featuring toxic chemicals, harsh operating conditions, and multiple time‐consuming steps, leading to long reaction times. Our innovative and universal approach offers an environmentally friendly, one‐pot, zero‐waste, and tunable route to functionalize solution‐processed TMDs via defect engineering. This straightforward method paves the way for the production of hybrid high‐performance materials tailored for multifunctional devices. Our systems serve as a versatile platform for various proof‐of‐concept applications, including photothermal and optical sensing, as well as electrocatalytic and electronic applications. These evaluations demonstrate the fundamental potential of our approach to create hybrid materials with significantly enhanced functionalities compared to pristine solution‐processed TMDs. By leveraging such defect‐engineered functionalization strategy, the systems exhibit superior performance across diverse applications, underscoring their versatility and providing the basis for further exploration of their properties and multifunctional devices.

## 2 Results and Discussion

### 2.1 Toposelective Functionalization via Galvanic Displacement

**Figure 2:**
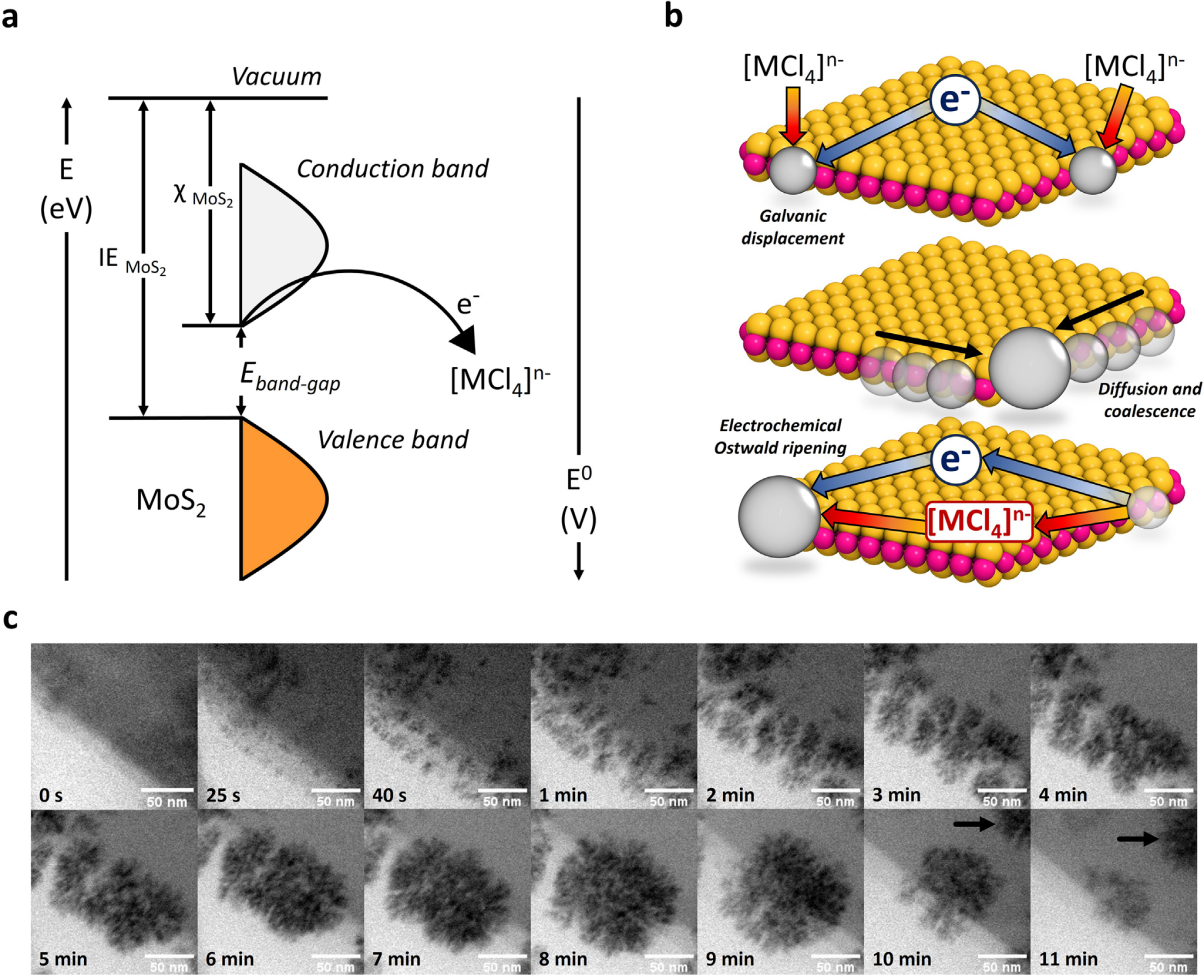
Galvanic displacement reaction between TMDs and [MCl_4_]^n−^. a) Energy diagram showing the optimal energy level matching between the semiconducting MoS_2_ and metal precursor [MCl_4_]^n−^, considering their energy level (left) and reduction potential (right). b) Sketch of the GD mechanism showing the three reaction steps leading to the growth of metal NPs at the flake edges (from top to bottom). c) In‐situ liquid STEM images collected at different time intervals during the GD reaction, showing the nucleation and growth of AuNPs on the MoS_2_ flake edges (the black arrows point to the electrochemical Ostwald ripening phenomena).

Colloidal dispersions of MS_2_ (with M = Mo, W) are produced by liquid‐phase exfoliation and analyzed following previously reported protocols (Sections S1 and S2, Supporting Information).^[^ ^15^ ^]^ MoS_2_ is taken as a case study for all characterizations and investigations, although the same rationale and findings apply to WS_2_ as well (Figure S2, Supporting Information). Starting from MoS_2_ dispersions (50 mL at 0.05 mg mL^−1^), 50 µL aliquots of aqueous HAuCl_4_ solutions (125 or 25 mM) are added dropwise under stirring (300 rpm) at a specific temperature (RT or 85 °C). Multiple additions are spaced by 15 min each, and the same synthesis protocol applies to all transition metal tetrachloride precursors ([MCl_4_]^n−^). The spontaneous and electroless GD reaction leads to MoS_2_ nanosheets functionalized with AuNPs (PdNPs or PtNPs in the case where K_2_PdCl_4_ and K_2_PtCl_4_ are used, respectively) selectively decorating the defect sites (i.e., flake edges), while the basal plane remains unaffected by the growth of metal NPs. In Section S2 (Supporting Information), we also systematically evaluate the seeded growth method, stoichiometric ratio between metal tetrachloride precursors and MoS_2_, as well as the influence of temperature. The stoichiometric ratio optimization is instrumental for achieving precise control over NP size and loading while maintaining edge selectivity. The golden rule guiding the redox GD reaction is an optimal energy level matching between the MoS_2_ electron affinity (χ) and the reduction potential (E_red_) of [MCl_4_]^n−^ (**Figure** **2**a),^[^ ^18^ ^]^ so that electrons are transferred from the semiconducting 2D materials (reducing agent, lower E_red_) to the metal precursors (oxidizing agent, higher E_red_), thus forming NPs (Section S3, Supporting Information).^[^ ^19^ ^]^ Owing to such a feature in the mechanism, we present an in‐situ protocol for the selective growth of metal NPs on the edges of TMDs, possessing significant advantages over traditional ex‐situ methods. Unlike the Turkevich approach (which requires separate AuNP synthesis, chemical functionalization, and then mixing), our method directly grows AuNPs on MoS_2_ defective sites, ensuring superior interfacial bonding and precise edge localization. The absence of reducing or capping agents enables the formation of NPs with bare surfaces, providing full accessibility during envisaged applications. The stoichiometry‐controlled reaction guarantees tunable size and loading, proceeding rapidly (<15 min) under mild conditions (25 °C, 1 atm). Our approach eliminates random NP distribution and tedious steps.

The driving force for such a toposelective reaction is the abundant presence of V_S_ at the edges of solution‐processed TMDs, playing a crucial role during all reaction steps (Figure 2b). In particular, V_S_ behave as reactive catalytic sites facilitating the adsorption of [MCl_4_]^n−^, followed by GD reaction to form metal nuclei (Section S4, Supporting Information). These nuclei then undergo diffusion and coalescence, primarily along the defective flake edges, forming larger clusters. Simultaneously, electrochemical Ostwald ripening occurs, during which some smaller metal clusters dissolve and promote the growth of larger clusters.^[^ ^18^ ^]^ The synergistic effects of these three steps (i.e., nucleation and growth, diffusion and coalescence, electrochemical Ostwald ripening) lead to the ultimate growth of metal NPs decorating the edge of MoS_2_ nanosheets. To investigate and validate the GD reaction mechanism, we perform in‐situ liquid scanning transmission electron microscopy (STEM) on MoS_2_ nanosheets, previously deposited on a suitable liquid‐phase TEM chip (Experimental Section) and then exposed to aqueous HAuCl_4_ solution (25 mM). Figure 2c displays bright field STEM images recorded at different time intervals, showing the nucleation and growth process of AuNPs at the flake edges. GD reaction, diffusion, and coalescence phenomena, as well as electrochemical Ostwald ripening (highlighted by the black arrows in Figure 2c), can be clearly distinguished, thereby confirming the proposed mechanism. It is worth mentioning that the dendritic morphology obtained for AuNPs during the in‐situ STEM study is induced by the electron beam, which makes fixation phenomena prevail over diffusion phenomena (at RT) above a threshold electron dose.^[^ ^20^ ^]^ Nevertheless, once a critical dimension is reached, dendritic clusters reshape into spherical NPs (Video S1, Supporting Information). When the GD reaction is carried out in the absence of the electron beam, spherical AuNPs are observed at the flake edges. This makes our approach even more advantageous for fine‐tuning the morphology of NPs and, thus, optimizing device performance. Our mechanism is further corroborated by density functional theory (DFT) calculations (Section S5, Supporting Information), where the presence of V_S_ entails a more favorable adsorption, dissociative, and desorption energy for the metal precursors on defective TMDs compared to pristine systems. The presence of V_S_ significantly favors the growth of metal NPs during the initial stages of the mechanism, where their formation energy results more favorable (viz., more negative) compared to pristine crystals.

Most importantly, Bader analysis reveals two electron‐transfer processes during the GD reaction: first, from MoS_2_ to HAuCl_4_, leading to the formation of AuNPs, and then from AuNPs to MoS_2_. Such a mechanism is expected to lead to negligible oxidation and doping effects on solution‐processed TMDs, as confirmed by the multiscale characterizations reported below. This is an additional unique feature of our functionalization protocol, as the majority of strategies reported in literature so far involve such side effects, influencing the material's properties and final device performance.

### 2.2 Multiscale Characterization of Selectively Edge‐Decorated TMDs with Metal NPs

**Figure 3:**
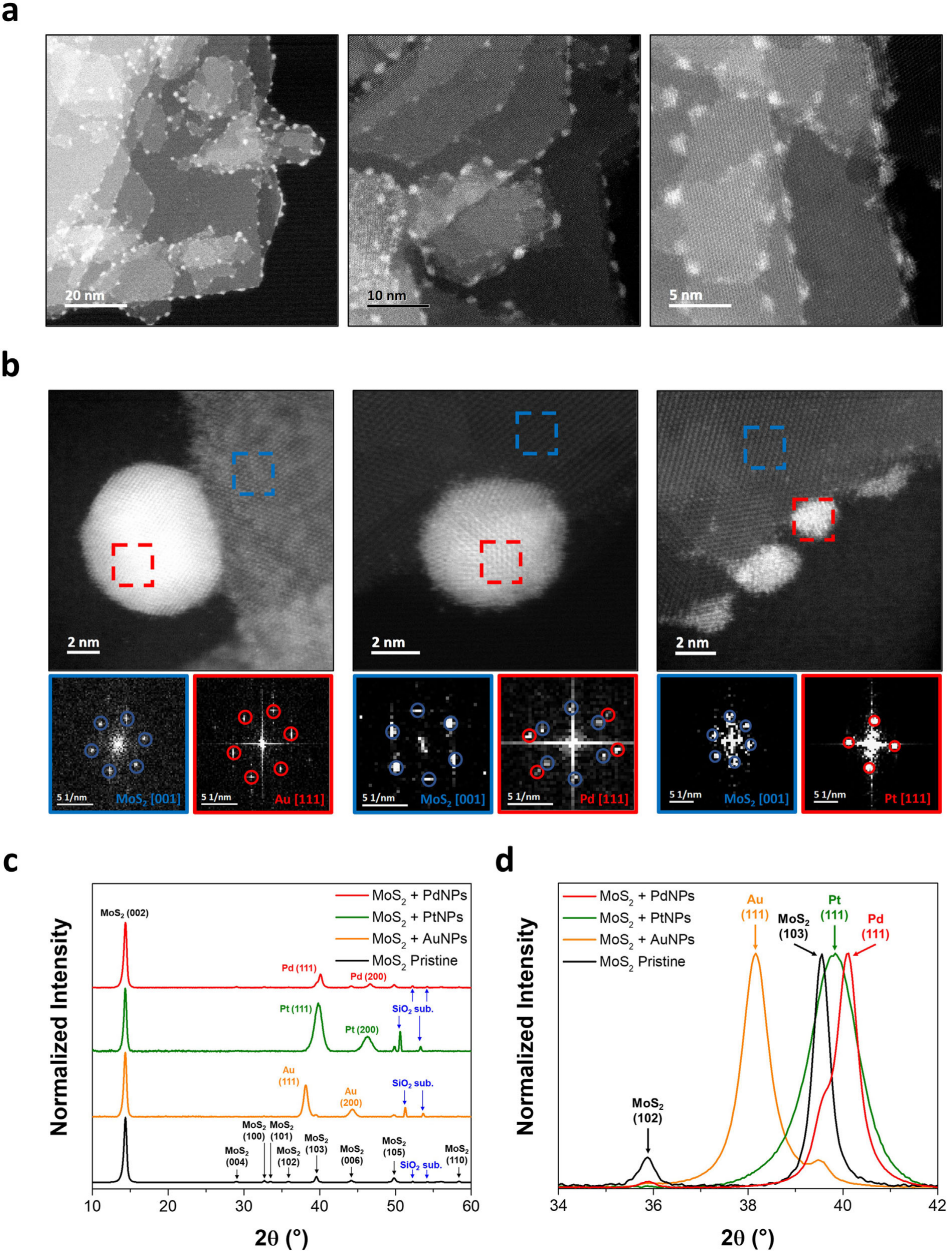
Morphological and structural characterization of MoS_2_ + metal NPs. a) Typical HAADF HR‐STEM images of edge‐decorated MoS_2_ + PtNPs nanosheets at increasing magnifications. b) HAADF HR‐STEM images (top) and related FFT patterns (bottom), obtained from the areas marked with red and blue squares, for MoS_2_ + AuNPs (left), MoS_2_ + PdNPs (middle), and MoS_2_ + PtNPs (right) nanosheets. c) GIXRD patterns for the MoS_2_ and three hybrids under investigation, along with a d) magnification on the region containing the (111) peak of the metal NPs.

We investigate the successful functionalization reaction via multiscale characterization techniques (**Figure** **3**; Section S5, Supporting Information). High‐resolution scanning transmission electron microscopy (HR‐STEM) was employed to map the morphology and distribution of metal NPs. As displayed in Figure 3a and Figure S12 (Supporting Information), regardless of the metal tetrachloride precursor employed, metal NPs selectively decorate the edges of MoS_2_ nanosheets. It is worth mentioning that when GD occurs at RT, we observe significant differences among the metal NPs. In particular, the size and abundance of AuNPs result in higher values compared to Pd and PtNPs, due to the different matching of energy levels which influence thermodynamic and kinetic features of the proposed mechanism. In particular, the Au precursor (viz., HAuCl_4_) shows a better energy level alignment (i.e., more suitable E_red_, see Section S3, Supporting Information) with the χ of MoS_2_, favoring the GD reaction. Conversely, K_2_PdCl_4_ and K_2_PtCl_4_ precursors exhibit a worse energy level matching, yielding to smaller cluster size and lower abundance for Pd and PtNPs. However, when the GD reaction for Pd and Pt precursors is performed at higher temperature (i.e., 85 °C), their density and size increase, corroborating the proposed energy‐activated mechanism (Figure S3, Supporting Information). Figure 3b displays typical high‐angle annular dark field (HAADF) HR‐STEM images for Au, Pd, and PtNPs decorating the edges of MoS_2_ nanosheets, along with the related fast Fourier transform (FFT) patterns obtained in different areas. The latter reveals six diffraction spots, in blue circles, characteristic of the (001)_MoS2_ crystal face, as well as the red circled diffraction spots from (111)_Au_, (111)_Pd_, and (111)_Pt_ orientations, confirming the crystalline nature of the metal NPs. This is in agreement with previous works, showing that low‐Miller‐index facets of face‐centered cubic (FCC) metals, with {111} having the lowest surface energy,^[^ ^21^ ^]^ grow with preferential orientation on the MoS_2_ basal plane.^[^ ^22^ ^]^ In our study, as a result of the GD reaction, metal NPs preferentially grow on defective sites, predominantly located at the edges of solution‐processed MoS_2_ nanosheets, providing favorable conditions for the nucleation and selective growth. The FFT results are also corroborated by grazing incidence X‐ray diffraction (GIXRD) analysis (Figure 3c,d) performed on thin films deposited on Si/SiO_2_ substrates. Typical MoS_2_ diffraction patterns are recorded along with the characteristic (111) and (200) peaks from the metal NPs, confirming their crystalline nature and structure. It is worth mentioning that GIXRD investigations, probing a larger sample area, reveal an additional (but minority) low‐Miller‐index facet compared to FFT results, namely the (200) peak. Most importantly, no signals from MoO_x_ species are recorded in the diffraction patterns, confirming the lack of oxidation reactions for MoS_2_ during the GD mechanism as a result of the two electron‐transfer processes mentioned above and confirmed by Bader analysis. Additionally, X‐ray photoelectron spectroscopy (XPS) analysis further confirms the lack of oxidation in MoS_2_ or WS_2_ upon GD reaction, in agreement with GIXRD results (Figure S13, Supporting Information).

### 2.3 Applications of Edge‐Decorated TMDs with Metal NPs

**Figure 4:**
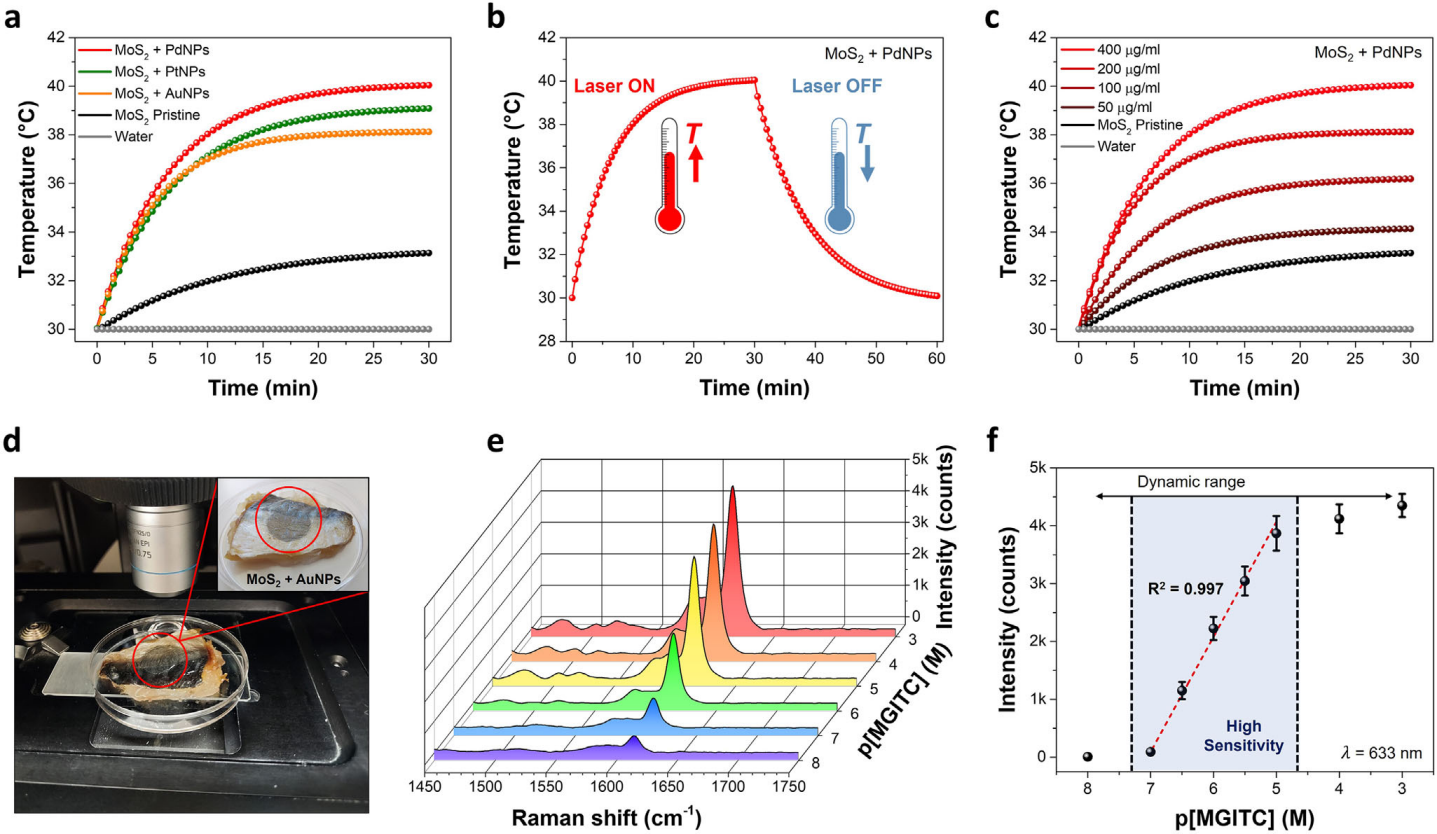
Photothermal and optical sensing via edge‐decorated MoS_2_ + metal NPs. a) Photothermal heating curves for MoS_2_ + metal NPs dispersions (400 µg mL^−1^), including pristine MoS_2_ nanosheets (400 µg mL^−1^) and water as a reference. b) Typical photothermal effect of MoS_2_ + PdNPs dispersion (400 µg mL^−1^), showing heating and cooling curve. c) Photothermal heating curves for MoS_2_ + PdNPs dispersions at different concentrations, showing pristine MoS_2_ nanosheets (400 µg mL^−1^) and water as a reference. All data reported in (a–c), are collected by using a 785 nm laser line with power density equal to 2 W cm^−2^. d) Experimental setup used to perform SERRS on a real‐world sample (fish) to detect MGITC at different concentrations. The sensing platform is represented by a thin film made of edge‐decorated MoS_2_ + AuNPs transferred on an ultrathin transparent PET substrate (highlighted by a red circle in the inset). e) 3D plot of SERRS spectra obtained for MGITC at different concentrations deposited on MoS_2_ + AuNPs/PET platform, along with the corresponding f) SERRS intensity (at 1616 cm^−1^) for MGITC at different concentrations, showing the range of maximum sensitivity. All data reported in (e) and (f) are collected using a 633 nm laser line.

**Figure 5:**
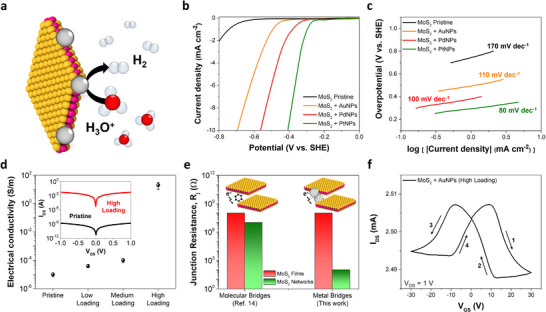
Electrocatalytic and electronics applications of edge‐decorated MoS_2_ + metal NPs. a) Sketch of the electrocatalytic activity toward HER for selectively edge‐decorated MoS_2_ + metal NPs. b) HER polarization curves for MoS_2_ + metal NPs as well as pristine MoS_2_ as a reference, along with c) corresponding Tafel plots. All experiments are performed in 0.5 m H_2_SO_4_. d) Electrical conductivity of spray‐coated thin films made of MoS_2_ + AuNPs as a function of AuNPs loading. The inset shows the typical output curves for pristine MoS_2_ and MoS_2_ + AuNPs (High loading) devices. e) Comparison of the junction resistance for MoS_2_ films and networks made via molecular or metallic bridging. f) Typical hysteresis loop in the transfer curves of MoS_2_ + AuNPs (High loading) devices, recorded by applying V_DS_ = +1 V and sweeping the V_GS_ from +30 to −30 V (direction highlighted by the arrows).

To evaluate our functionalization strategy, we test the resulting hybrid materials for various proof‐of‐concept applications (**Figures** **4** and **5**; Section S6, Supporting Information). First, we use aqueous MoS_2_ + metal NPs dispersions to assess their performance for photothermal detection. To ensure consistency and opportunity for comparison, MoS_2_ concentration is kept constant (400 µg mL^−1^) while we evaluate the performance upon functionalization with different metal NPs. To achieve comparable size and loading of the metal NPs, one addition of 125 mM metal tetrachloride precursor is used, with the reaction performed at RT for AuNPs and at 85 °C for PdNPs and PtNPs. To explore the potential use of such systems in the first biological window (from 700 to 950 nm), a 785 nm laser line (power density = 2 W cm^−2^) is employed. The temperature profiles shown in Figure 4a point out that MoS_2_ + NPs, regardless of the metal, outperform pristine MoS_2_. Among the samples, MoS_2_ + PdNPs exhibits the highest temperature increase, attributed to the superior thermal stability, strong light absorption, and light‐to‐heat conversion efficiency of PdNPs. This is followed by MoS_2_ + PtNPs and MoS_2_ + AuNPs, while pristine MoS_2_ and water show minimal heating effects. From the typical heating and cooling curves (Figure 4b), not only we demonstrate the reversible nature of the photothermal behavior of these hybrid systems, but we also calculate the photothermal conversion efficiency for all samples (Section S6, Supporting Information), showing the following order: MoS_2_ + PdNPs (55%) >MoS_2_ + PtNPs (50%) > MoS_2_ + AuNPs (45%) > pristine MoS_2_ (15%). Additionally, Figure 4c displays the concentration‐dependent performance of MoS_2_ + PdNPs, showing that higher concentrations result in larger temperature variations, with the 400 µg mL^−1^ sample exhibiting the best performance within the concentration range under investigation.

These findings highlight a significant enhancement of the photothermal conversion efficiency for the hybrid systems, paving the way for a large variety of applications.^[^ ^23^ ^]^ By the same token, we assess the performance of edge‐decorated MoS_2_ + metal NPs systems for optical sensing via surface‐enhanced resonance Raman scattering (SERRS) spectroscopy. To this end, we use MoS_2_ + AuNPs samples, considering that only AuNPs show plasmonic properties within the visible and the near‐infrared range, commonly explored for practical applications, unlike Pd and PtNPs, whose plasmons typically fall in the deep‐UV range. Then, we fabricate hybrid plasmonic platforms made of MoS_2_ + AuNPs thin films and (dry) transfer them onto ultrathin (1.4 µm thick) polyethylene terephthalate (PET) substrates (Experimental Section and Section S6, Supporting Information). We investigate the SERRS properties of these samples by using malachite green isothiocyanate (MGITC) model analyte, well‐known to be a toxic and hazardous chemical to humans, but still commonly used in aquaculture as a parasiticide (Figure S15, Supporting Information). MGITC shows a strong absorption band ≈620 nm (in water) due to n→π* electronic transition.^[^ ^24^ ^]^ To leverage this property, we employ a Raman set‐up equipped with a 633 nm laser line, ensuring that the excitation laser wavelength closely matches the electronic transition wavelength of the analyte. This resonance condition significantly enhances sensitivity, enabling SERRS. Additionally, we assess the performance of the optimized hybrid plasmonic platform to detect MGITC in a real‐world fish sample (Figure 4d), exploring a broad concentration range (expressed as p[MGITC] = – log[MGITC]) from 10 nM to 1 mM. It is worth noting that our hybrid sensing platforms show a remarkable SERRS activity (Figure 4e) with a high sensitivity from 100 nM to 10 µM (≈2000 counts/log[M]), obtained from the slope of the linear trend shown in Figure 4f, which represents the concentration range of interest to meet the relevant European guidelines. Therefore, our hybrid MoS_2_ + AuNPs systems demonstrate high SERRS activity, making them highly suitable for real‐world applications, such as detecting hazardous analytes in complex environments.

We also investigate the performance of devices based on selectively edge‐decorated MoS_2_ nanosheets with metal NPs for their potential application in electrocatalysis and electronics. The presence of noble metal centers provides highly catalytic active sites and enhances hydrogen evolution reactions (HERs) performance, leading to higher electrocatalytic activity than pristine MoS_2_ nanosheets (**Figure** **5**a). To this end, commercial screen‐printed carbon electrodes are coated with the hybrid materials under analysis by drop casting (keeping constant the sample amount and size of NPs, for fair comparison), using 0.5 m H_2_SO_4_ as acidic electrolyte to test their electrocatalytic activity (Experimental Section). The HER polarization curves (recorded from 0 to −0.8 V vs. standard hydrogen electrode, SHE) displayed in Figure 5b highlight a dramatic decrease of the overpotential triggering HERs for MoS_2_ + metal NPs systems. In particular, in terms of activity, we observe MoS_2_ + PtNPs > MoS_2_ + PdNPs > MoS_2_ + AuNPs > pristine MoS_2_, thereby confirming the superior electrocatalytic performance of noble metals toward HERs along with their trend, usually reported in well‐known “volcano plots” based on thermodynamic calculations (Video S2, Supporting Information).^[^ ^25^ ^]^ Moreover, the corresponding Tafel plots displayed in Figure 5c corroborate the enhanced electrocatalytic activity of MoS_2_ + metal NPs systems, as shown by their significantly smaller Tafel slope compared to pristine MoS_2_. Such a difference denotes a drastic change in the HER electrochemical kinetics and reaction mechanism: while MoS_2_ + PdNPs, MoS_2_ + AuNPs and pristine MoS_2_ point to a Volmer–Heyrovsky mechanism (Volmer: H^+^ + e^−^ → H_ad_; Heyrovsky: H_ad_ + H_2_O + e^−^ → H_2_ + OH^−^) due to a Tafel slope close to 120 mV dec^−1^ (theoretical value), MoS_2_ + PtNPs suggest a Volmer–Tafel mechanism (Volmer: H^+^ + e^−^ → H_ad_; Tafel: 2 H_ad_ → H_2_) by reason of a Tafel slope close to 40 mV dec^−1^ (theoretical value).^[^ ^26^ ^]^ These results underscore the significant potential of MoS_2_ + metal NPs for HERs in acidic electrolyte, while also highlighting the opportunity for further enhancement of their electrocatalytic activity in future studies by fine‐tuning the size, loading, and shape of decorating NPs.

Finally, we assess the performance of our hybrid systems when used as active materials for electronic applications, running 2‐ and 3‐terminal device measurements and using MoS_2_ + AuNPs as a case study. In particular, we produce three different batches (Experimental Section) with increasing loading of NPs at the flake edges (referred to as low, medium and high loading, respectively), taking advantage of the inherent features of the GD reaction and stoichiometry‐controlled growth mechanism discussed above (viz., increased NP loading when increasing the number of additions for the metal precursors). The electronic devices are prepared by spray coating the aqueous dispersions of MoS_2_ + AuNPs on Si/SiO_2_ substrates, followed by gold electrode deposition (Experimental Section). Figure 5d displays an increase in the electrical conductivity while increasing the loading of AuNPs, with a dramatic boost by almost six orders of magnitude in the case of high‐loading samples. This phenomenon is characteristic of percolative systems and is typically observed when the percolation threshold is reached.^[^ ^16^ ^]^ In this case, the striking increase in electrical conductivity in high‐loading samples is likely due to the formation of networks made of MoS_2_ nanosheets bridged together by metal NPs decorating their edges. These metal bridges improve the overall charge carrier transport in the hybrid semiconducting materials under investigation and lead to a drastic reduction of their inter‐flake junction resistance (R_J_) by six orders of magnitude (Figure 5e). The change of electrical conductivity matching that of R_J_ is not coincidental. From 3‐terminal device measurements (i.e., transistor geometry) performed on high‐loading MoS_2_ + AuNPs samples, we observe a major enhancement in the main figures of merit compared to pristine MoS_2_ devices. In particular, from the transfer curves (Figure 5f), obtained by sweeping the gate voltage (V_GS_) from +30 to −30 V at a fixed source–drain voltage (V_DS_) equal to +1 V, we calculate a field‐effect mobility of ≈1 cm^2^ V^−1^ s^−1^ for the high‐loading sample, being higher than typical pristine MoS_2_ devices by a factor 10^6^ (Experimental Section).^[^ ^15^ ^]^ Considering the electrical conductivity of the network expressed as σ_*Net*_ = *n_Net_* *e*µ_*Net*_, with *n_Net_* representing the network charge carried density, µ_*Net*_ the network mobility and *e* the elementary charge, as well as assuming *R_J_* ∝1/µ_*Net*_ according to recently developed models,^[^ ^27^, ^28^ ^]^ we infer that the change of σ_*Net*_ in MoS_2_ + AuNPs samples is primarily ascribed to a change in the µ_*Net*_. This is further corroborated by the unaltered charge carrier density (viz., no doping effect) of MoS_2_ devices upon decoration with metal NPs by GD, due to its inherent mechanistic features highlighted above. To the best of our knowledge, these findings make our functionalization strategy the most effective route to boost the electrical performance of solution‐processed TMD‐based devices thus far, characterized by an improved electrical conductivity, mobility, as well as inter‐flake junction resistance by six orders of magnitude, without resorting to more complex, time‐consuming, and unscalable methods. Such a protocol holds great potential to develop innovative technologies for real‐world applications, including printable and wearable electronics.

Also, from the transfer curves of MoS_2_ + AuNPs (high loading) reported in Figure 5f, we observe a significant hysteresis loop along with the presence of negative transconductance (*dI*_DS_/*dV*_GS_ < 0), both responsible for the characteristic “butterfly” curve. This is not common for pristine MoS_2_ devices, and it is likely due to the presence of metal NPs at the flake edges affecting the charge transport properties and mechanisms. This behavior deserves further investigation in future studies because it could pave the way for the potential use of such systems for memory devices.

## 3 Conclusion

We report on a straightforward and universal functionalization protocol for solution‐processed TMDs via defect engineering. We capitalize on the GD reaction between TMDs and transition metal tetrachloride complexes to produce selectively edge‐decorated nanosheets with Au, Pd, or PtNPs, whose size and loading can be tuned on demand by a stoichiometry‐controlled growth. The resulting hybrid materials exhibit significantly higher performance than pristine TMDs for photothermal and optical sensing, as well as electrocatalytic HERs and electronic applications. Such a toposelective functionalization approach represents an alternative green, zero‐waste, fast, and versatile route to produce high‐performance multifunctional devices for real‐world applications. Future experimental and theoretical investigations are needed to extrapolate and calculate thermodynamic and kinetic parameters for various 2D materials and metal precursors. Therefore, our strategy provides ample room for future investigations, in which the main focus could be on i) expanding the list of reacting TMDs and metal precursors, ii) fine‐tuning the morphological and chemical properties of metal NPs, as well as their (iii) influence on device performance.

## 4 Experimental Section

### In‐Situ STEM Analysis to Study Nucleation and Growth of NPs via GD Mechanism

MoS_2_ + AuNPs samples are considered a case study. The Poseidon liquid holder by Protochips Inc. was used. The holder was equipped with the commercially available flow liquid cell (Protochips Inc.), consisting of a bottom E‐chip and a top E‐chip spaced 150 nm apart. Both chips were equipped with an observation window (550 × 50 µm^2^) covered by a 30 nm‐thick amorphous Si_3_N_4_ film. To make the chip surface more hydrophilic, first, they were exposed to plasma cleaning (Ar and O_2_) for 20 s. Then, a 2 µL aliquot of MoS_2_ dispersion (2 mg L^−1^) was drop cast onto the bottom E‐chip placed in parallel configuration with the Si_3_N_4_ window. Later, the aqueous HAuCl_4_ solution (25 mM) was allowed to flow inside the liquid cell at 50 µL min^−1^ flow rate through the inlet of the holder. When the liquid reached the observation windows, the flow was stopped, and the investigation was carried out in static mode. The nucleation and growth processes of AuNPs were observed in STEM mode using a spherical aberration‐corrected JEOL 2100F TEM microscope, operated at 200 kV and equipped with bright‐ and dark‐field detectors. The investigations were carried out with a probe size of 0.13 nm and a current of 86 pA. The data, in particular STEM images (512 × 512 pixels and 8 µs of dwell time), were continuously recorded with the AXON in‐situ TEM software by Protochips Inc.

### Potential Applications of Edge‐Decorated TMD Nanosheets with Metal NPs—Photothermal Sensing

A Raman Renishaw InVia spectrometer was used to this end, equipped with a 785 nm laser line (power = 260 mW, spot size = 4 mm in diameter, power density = 2 W cm^−2^). 2 mL of a MoS_2_ + metal NPs dispersion was transferred inside a quartz cuvette. Before running the analysis (keeping the dispersion under stirring), the cuvette was covered with Styrofoam (equipped with a hole to let the laser pass through) to avoid thermal dissipation from the quartz container. The changes in temperature during laser irradiation were recorded by using a digital thermocouple immersed in the dispersion. The same setup and operating conditions were used for all samples.

### Potential Applications of Edge‐Decorated TMD Nanosheets with Metal NPs—Optical Sensing

A Raman Renishaw InVia spectrometer was used for this purpose, equipped with a 633 nm laser line with a 50× objective (N.A. 0.75), 0.33 mW of maximum power, and 1 s acquisition time. Hybrid plasmonic platforms are prepared as follows: i) MoS_2_ + AuNPs aqueous dispersions (400 µg mL^−1^) were vacuum filtered by using hydrophilic polytetrafluoroethylene membranes (25 mm in diameter, pore size = 0.1 µm); ii) before the complete dry‐up of the membrane, it was stamped on ultrathin PET (1.4 µm thick) substrate and, with the help of a manual laboratory bench press, iii) it was kept pressed for 30 min. After that, the press was opened, and the dry‐transfer process on the PET film was successful. To optimize the SERRS performance, three different loadings of AuNPs on MoS_2_ were explored, corresponding to small (1 addition), medium (3 additions), and high (5 additions). In the three cases, the concentration of the stock HAuCl_4_ solution was 125 mM, and the reactions were performed at RT. To assess the SERRS performance of the different hybrid plasmonic platforms, 10 µL of 10^−5^ m MGITC was drop cast on the substrates and dried before SERRS measurements. Then, 441 points were randomly measured across the substrates, and the average signal intensity and standard deviation were calculated.

For testing the platforms on the real‐world sample, the MGITC‐coated substrates were placed on fish skin, with measurements collected upside down. This inverted configuration was made possible by the ultrathin PET substrate, which facilitated light transmission and sample handling.

### Potential Applications of Edge‐Decorated TMD Nanosheets with Metal NPs—Electrocatalytic HERs

Such an investigation was performed on an Autolab PGSTAT128N instrument (Metrohm) under ambient conditions. Samples were prepared by drop casting (20 µL) the MoS_2_ + metal NPs dispersions (400 µg mL^−1^) onto commercial screen‐printed carbon electrodes, showing working (diameter = 0.40 cm) and auxiliary electrodes made of carbon, while the reference electrode was made of Ag/AgCl (Metrohm DropSens–110). For all samples, the stoichiometric ratio between MoS_2_ and metal precursor was kept constant and equal to 1:1. The acidic electrolyte used is 0.5 m H_2_SO_4_, with a total volume of ≈50 µL necessary to wet all three electrodes. The linear sweep voltammetry curves were recorded from 0 to −0.8 V vs. SHE, with a scan rate equal to 4 mV s^−1^. For each sample, the polarization curves were collected three to five times.

### Potential Applications of Edge‐Decorated TMD Nanosheets with Metal NPs—Electronics

The electrical characterization (both 2‐ and 3‐terminal devices) of pristine MoS_2_ and MoS_2_ + AuNPs thin films (same materials showing different AuNP loading, as for the optical sensing) was carried out under ambient conditions using a Keithley 2612A connected to a Suss probe station. Top contacts, made of 100 nm‐thick Au, were deposited onto the samples (spray‐coated onto Si/SiO_2_ substrates) using a Temescal FC2000 metal evaporator. The thickness of the samples was measured using a Profilm3D Optical Profiler (Filmetrics) operating in white‐light interferometry mode with a 50x Nikon DI objective lens. From 3‐terminal (i.e., back‐gated transistor geometry) measurements and the related transfer curves, the field‐effect mobility (µ_FE_) is calculated as follows:

(1) $$\mu_{\mathrm{FE}}=\left(\frac{dI_{\mathrm{DS}}}{dV_{\mathrm{GS}}}\right)\frac{L}{WV_{\mathrm{DS}}C_{\mathrm{ox}}}\;\;\;$$

where *I*_DS_ and *V*_GS_ represent the source–drain current and gate‐source voltage, respectively, *L* and *W* are the channel length and width (50 µm and 18 mm, respectively), *V*_DS_ is the source–drain voltage, and *C*_ox_ is the oxide capacitance (≈11 nF cm^−2^ in case of 300 nm‐thick SiO_2_).

Moreover, to calculate and compare *R_J_* for pristine MoS_2_ films and MoS_2_ + AuNPs networks, the following equations are used:^[^ ^27^, ^28^ ^]^

(2) $$\sigma_{Film/Network}\approx \left(1-P\right)n_{NS}l_{NS}^{2}/4R_{J}k$$

(3) $$\mu_{Film/Network}\approx l_{NS}^{2}/eR_{J}$$

where *P* and *k* represent the film/network porosity and tortuosity, respectively, *e* the elementary charge, *n_NS_* and *l_NS_* describing the nanosheet charge carrier density and lateral size, respectively.

Further details about materials, characterization techniques, and data treatment can be found in the Supporting Information.

## Conflict of Interest

The authors declare no conflict of interest.

## Author Contributions

S.I., V.M.G., and P.S. conceived the experiments and designed the study. V.M.‐G. contributed to the sample preparation and to the SERRS measurements. A.G.K. and J.N.C. produced the MoS_2_/WS_2_ dispersions and performed the electrical characterizations. V.G.C., W.B., and O.E. carried out the in‐situ liquid TEM analysis. M.J.C.‐F., J.P.‐J., and I‐P.‐S. performed the SERS investigations. A.D. and G.C. performed the DFT calculations. All authors discussed the results and contributed to data interpretation. S.I. and P.S. co‐wrote the paper with input from all co‐authors.

## Supporting information



Supporting Information

In situ Liquid TEM MoS2 AuNPs

HERs MoS2 PtNPs

## Data Availability

The data that support the findings of this study are available from the corresponding author upon reasonable request.
