# Supplementary material for: Toposelective Functionalization of Solution‐Processed Transition Metal Dichalcogenides with Metal Nanoparticles via Defect Engineering
Source: Adv Mater. 2025 Aug 16;37(43):e06605. doi: 10.1002/adma.202506605 (PMC12574622; doi:10.1002/adma.202506605)
Supplement: Supplementary file 1 — Supporting Information [file ADMA-37-e06605-s002.docx]

Supplementary Information

**Toposelective functionalization of solution-processed transition metal dichalcogenides with metal nanoparticles via defect engineering**

Stefano Ippolito^a^, Verónica Montes-García^a^, Adam G. Kelly^b^, Valentina Girelli Consolaro^c^, Walid Baaziz^c^, María José Cordero-Ferradás^d^, Arezoo Dianat^e^, Jorge Pérez-Juste^d^, Isabel Pastoriza-Santos^d^, Ovidiu Ersen^c^, Gianaurelio Cuniberti^e^, Jonathan N. Coleman^f^, Paolo Samorì^a,^*

^a^ Université de Strasbourg, CNRS, ISIS UMR 7006, 8 allée Gaspard Monge, F-67000 Strasbourg, France
^b^ i3N/CENIMAT, Faculty of Science and Technology, Universidade NOVA de Lisboa, Campus de Caparica, 2829-516 Caparica, Portugal

^c^ Université de Strasbourg, CNRS, IPCMS, 23 rue du Loess, BP 43, 67034 Strasbourg Cedex 2, France

^d^ CINBIO, Universidade de Vigo, Campus Universitario Lagoas-Marcosende, 36310 Vigo, Spain

^e^ Institute for Materials Science and Max Bergmann Center of Biomaterials, TU Dresden, 01062 Dresden, Germany

^f^ School of Physics, Centre for Research on Adaptive Nanostructures and Nanodevices (CRANN) and Advanced Materials and Bioengineering Research (AMBER), Trinity College Dublin, Dublin 2, Ireland

* Corresponding author: [samori@unistra.fr](mailto:samori@unistra.fr)

**Table of Contents**

1. Raw materials and characterization techniques…………………………………………………….S2
2. Synthesis optimization of edge-decorated TMDs with metal NPs………..………………………S4
3. Galvanic displacement mechanism...…………………………………………………………….….S9
4. Density functional theory (DFT) calculations……………………………………………………….S13
5. Multiscale characterization of edge-decorated TMDs with metal NPs………………………….S16
6. Applications of edge-decorated TMDs with metal NPs…………………………………………....S19
7. Captions for the supplementary videos ………………..…………………………………………..S24
8. References…………………………………………………………………………………………….S24
9. **Raw materials and characterization techniques**

MoS_2_ and WS_2_ powders (purity ≥99%) are sourced from Sigma Aldrich. Tetrachloroauric(III) acid, potassium tetrachloroplatinate(II), and potassium tetrachloropalladate(II) are sourced from Sigma Aldrich. All chemicals are used without further purification steps.

Spectroscopic characterizations

- UV-Vis absorption spectra are recorded on a JASCO v670 spectrometer using quartz cuvettes (optical path = 10 mm) under ambient conditions.
- Raman and SERRS measurements are conducted with a Renishaw InVia Reflex system. The spectrograph used a high-resolution grating (1200 grooves cm^−1^) with additional band-pass filter optics, a confocal microscope, and a 2D-CCD camera. Laser excitation is carried out at 633 nm with a 50× objective (N.A. 0.75), 0.33 mW of maximum power, and 1 s acquisition time.
- X-ray photoelectron spectroscopy (XPS) is performed with a Thermo Scientific K-Alpha X-ray photoelectron spectrometer, operating with a base chamber pressure ~10^-9^ mbar and an Al anode as X-ray source (Al_Kα_ radiation = 1486.6 eV). The X-ray beam spot size is ~400 µm. Peak fitting is performed with constraints on the full width half maximum (FWHM) and peak area ratio of the spin-orbit components, as well as their peak position.
- Photoelectron spectroscopy in air (PESA) analysis is performed with a Riken Keiki AC-2 under ambient conditions, with energy step of 0.05 eV, a UV spot intensity of 10 nW, within an energy range of 3.4 to 6.2 eV.

Morphological, structural and surface characterizations

- Scanning electron microscopy (SEM) and scanning transmission electron microscopy (STEM) are performed with a Quanta FEG 450, operating with a chamber pressure ~10^-6^ torr and gun pressure ~10^-9^ torr, working at 20-30 kV.
- In-situ STEM measurements are performed with an electron probe of 0.13 nm and a beam current of 86 pA. BF- and DF-HR-STEM images (1024 x 1024 pixels) are taken with a dwell time of 15 µs (camera length of 12 cm). Typical TEM copper grids with a holey carbon film are used.
- Grazing incidence X-ray diffraction (GIXRD) experiments are performed using a Bruker D8 Advanced with twin-twin optics. The diffraction patterns are recorded in a 2θ range from 10° to 60°, using an angle of incidence equal to 0.5° and Cu_Kα_ radiation (λ = 1.5418 Å) at 40 kV and 40 mA. The data are acquired with a step of 0.04° and a total scan time of 20 hours.
- The thickness of the films is measured using a Profilm3D Optical Profiler (Filmetrics) operating in white-light interferometry (WLI) mode with a 50× Nikon DI objective lens.

Electrical and electrochemical characterizations

- The electrical characterization is carried out under ambient conditions using a Keithley 2612A connected to a Suss probe station. Top-contact gold electrodes (with thickness equal to 100 nm) are deposited using a Temescal FC2000 metal evaporation system
- Cyclic voltammetry (CV) and linear sweep voltammetry (LSV) are performed using an Autolab PGSTAT128N instrument (Metrohm) under ambient conditions. Commercial screen-printed carbon electrodes (Metrohm DropSens) are used, having working and auxiliary electrodes made of carbon, while reference electrode is made of Ag/AgCl. The electrolyte used is 0.5 M H_2_SO_4_.

1. **Synthesis optimization of edge-decorated TMDs with metal NPs**

Production of TMDs via liquid-phase exfoliation

The MoS_2_ and WS_2_ inks are prepared by ultrasonicating the bulk powders in 2-propanol (IPA). An initial concentration of 20 mg/mL is processed for 1 hour in 80 ml of IPA using a tip horn sonicator (Sonics Vibra-cell VCX-750 ultrasonic processor) at 50 % amplitude with an ON:OFF pulse of 6:2. The resulting dispersion is centrifuged at 3218 g for 1 hour using a Hettich Mickro 220R, after which the entirety of the supernatant is discarded to remove potential contaminants from the starting powders. The sediment is then redispersed in 80 mL of fresh IPA and sonicated for 6 hours at 50 % amplitude with an ON:OFF pulse of 4:4. This process results in a polydisperse stock dispersion from which nanosheets can be size-selected by liquid-cascade centrifugation. The polydisperse stock is first centrifuged at 106.4 g for 90 minutes to remove the largest aggregates. The top 90 % of the supernatant is then centrifuged at 425.6 g for 90 minutes. Then, the supernatant containing the small nanosheets is completely decanted from the vial and the sediment is redispersed in 30 mL of IPA to give the final dispersion.

Synthesis of selectively edge-decorated TMD nanosheets with noble metal NPs

Starting from TMD dispersions in IPA with concentration ≈ 2 mg/mL, 1 mL is diluted in 50 mL of deionized water. Under continuous stirring (300 rpm) and a specific temperature (room temperature or 85 ºC), 50 µL aliquots of transition metal tetrachloride (*i.e.*, HAuCl_4_, K_2_PdCl_4_, or K_2_PtCl_4_) aqueous solutions (25 or 125 mM) are added. The number of aliquots is determined by the targeted stoichiometric ratio between the 2D material and metal tetrachloride precursor, with each addition spaced at 15-minute intervals. After synthesis, the resulting dispersions, made of edge-decorated TMD nanosheets with noble metal NPs, are centrifuged at 10,000 rpm for 10 minutes (to precipitate the whole amount of material) and then, upon removing the supernatant, redispersed in water. Such centrifugation steps are repeated 3 times in total. After the last one, the precipitate is redispersed in water according to the desired concentration needed during the following characterization steps or sample preparation.

Optimization of synthesis parameters


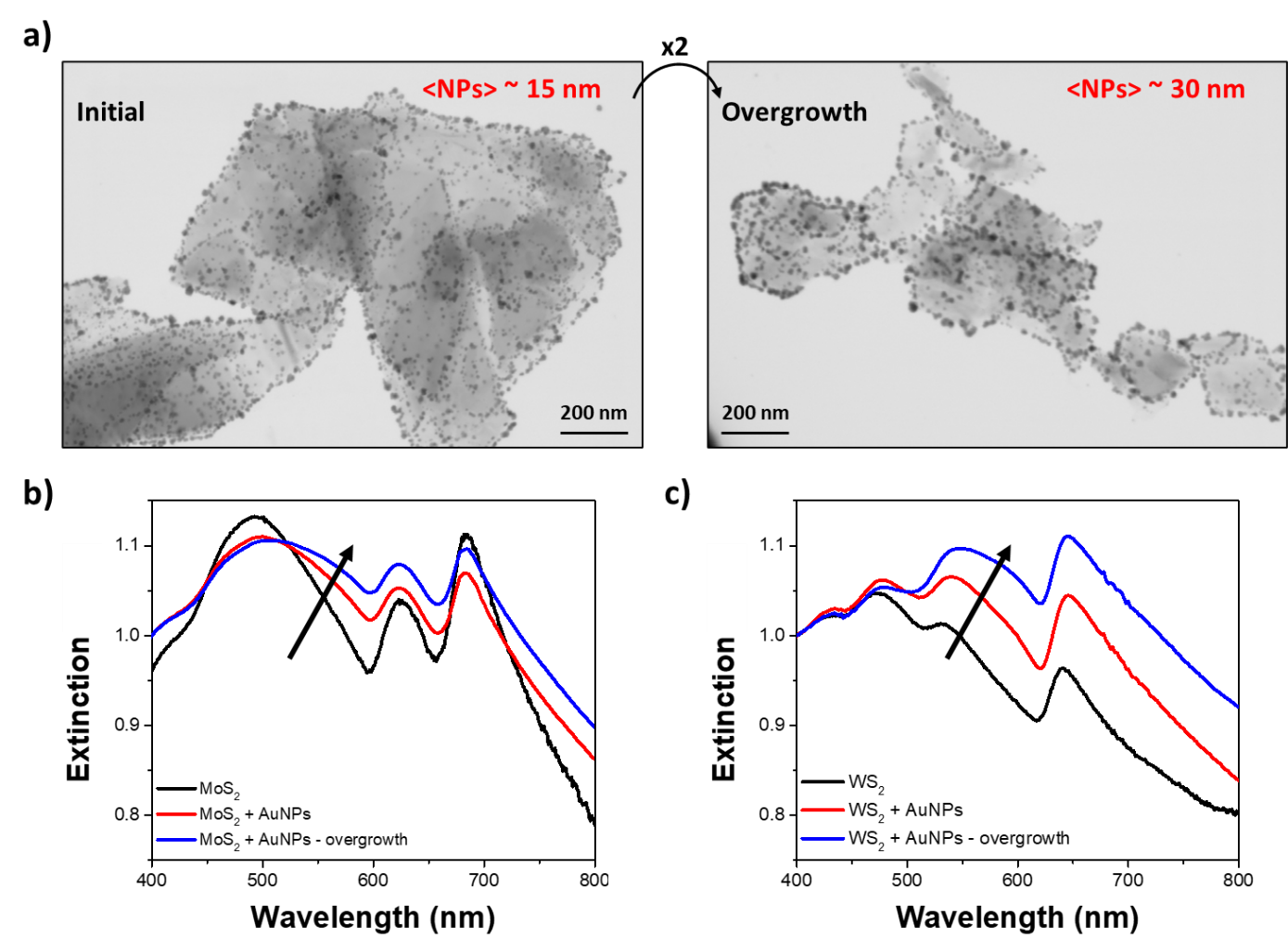


**Supplementary Fig. 1 | Seeded growth method for edge-decorated TMDs + metal NPs.** **a**, STEM images of MoS_2_ + AuNPs synthesized via seeded-growth method. **b**, Extinction spectra for MoS_2_, MoS_2_ + AuNPs and MoS_2_ + AuNPs – overgrowth. **c**, Extinction spectra for WS_2_, WS_2_ + AuNPs and WS_2_ + AuNPs – overgrowth. The syntheses are performed at room temperature with a 125 mM stock aqueous solution of HAuCl_4_.

Our initial step for the synthesis optimization involves growing metal NPs on TMDs by using a seeded growth method. This approach entails the sequential addition of HAuCl_4_ to MoS₂, allowing controlled and selective particle growth at the flake edges. As shown in Supplementary Fig. 1a (left), the addition of a single HAuCl_4_ aliquot (125 mM) leads to the formation of small AuNPs (≈15 nm) selectively localized at the MoS₂ edges. By repeating the HAuCl_4_ addition twice, the resulting AuNPs show an average size twice (≈30 nm) as large as those obtained by a single addition, as displayed in Supplementary Fig. 1a (right). The seeded growth method is also applied to WS₂ and HAuCl_4_, yielding comparable results.

Supplementary Fig. 1b and Fig. 1c display the extinction spectra of MoS₂ and WS₂, respectively, before and after the growth of AuNPs, as well as upon overgrowth of the latter. In Supplementary Fig. 1b, the extinction spectrum of MoS₂ shows the characteristic excitonic peaks, due the electronic transitions in the material. Upon the growth of AuNPs, a plasmonic peak appears (λ_AuNPs_ ≈ 520 nm, highlighted by the black arrow), indicating the successful growth of AuNPs on MoS₂. Following the overgrowth of the AuNPs (*viz*., seeded-growth method), the plasmonic peak redshifts and broadens, as a result of the increased NP size as well as potential changes in their local environment (*e.g.*, enhanced coupling between adjacent particles). In Supplementary Fig. 1c, the extinction spectrum of WS₂ shows the typical excitonic features. Similar to MoS₂, the growth of AuNPs on WS_2_ results in the appearance of a plasmonic peak around 520 nm. After the overgrowth step, the plasmonic peak broadens and redshifts, due to the growth of larger AuNPs. These effects, observed for both MoS₂ and WS₂, underscore the tunability of their optical response by changing the size and distribution of AuNPs, which could be leveraged for sensing and catalysis applications.


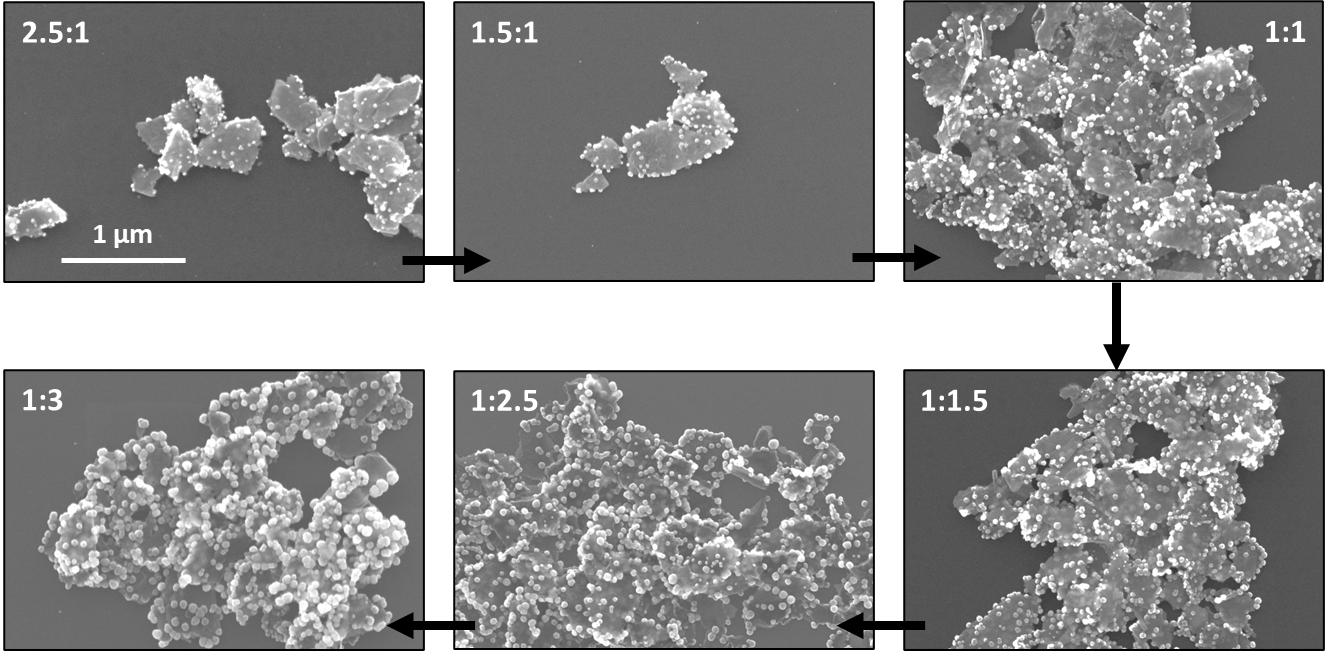


**Supplementary Fig. 2 | Stoichiometry-controlled growth of metal NPs.** SEM images of MoS_2_ + AuNPs taken on a sample with decreasing MoS_2_:HAuCl_4_ stoichiometric ratio. The synthesis is performed at room temperature by sequential additions of 125 mM stock aqueous solution of HAuCl_4_.

As a next step, the influence of different stochiometric ratios between MoS₂ and HAuCl_4_ is explored, achieved by using the seeded growth method described above. The stoichiometric ratio between the two reagents is critical, as it governs the nucleation and growth dynamics of the AuNPs at the MoS₂ flake edges, in addition to the overall uniformity, stability, and functionality of the final hybrid material. As shown in Supplementary Fig. 2, the increasing HAuCl_4_:MoS_2_ stoichiometric ratio (*viz.,* increasing number of HAuCl_4_ sequential additions) leads to a corresponding increase in both size and loading of AuNPs. However, as the amount of HAuCl_4_ continues to increase and the edges become fully decorated, the toposelective nature of the reaction is undermined and the AuNPs start growing on the (less reactive) basal planes as well.


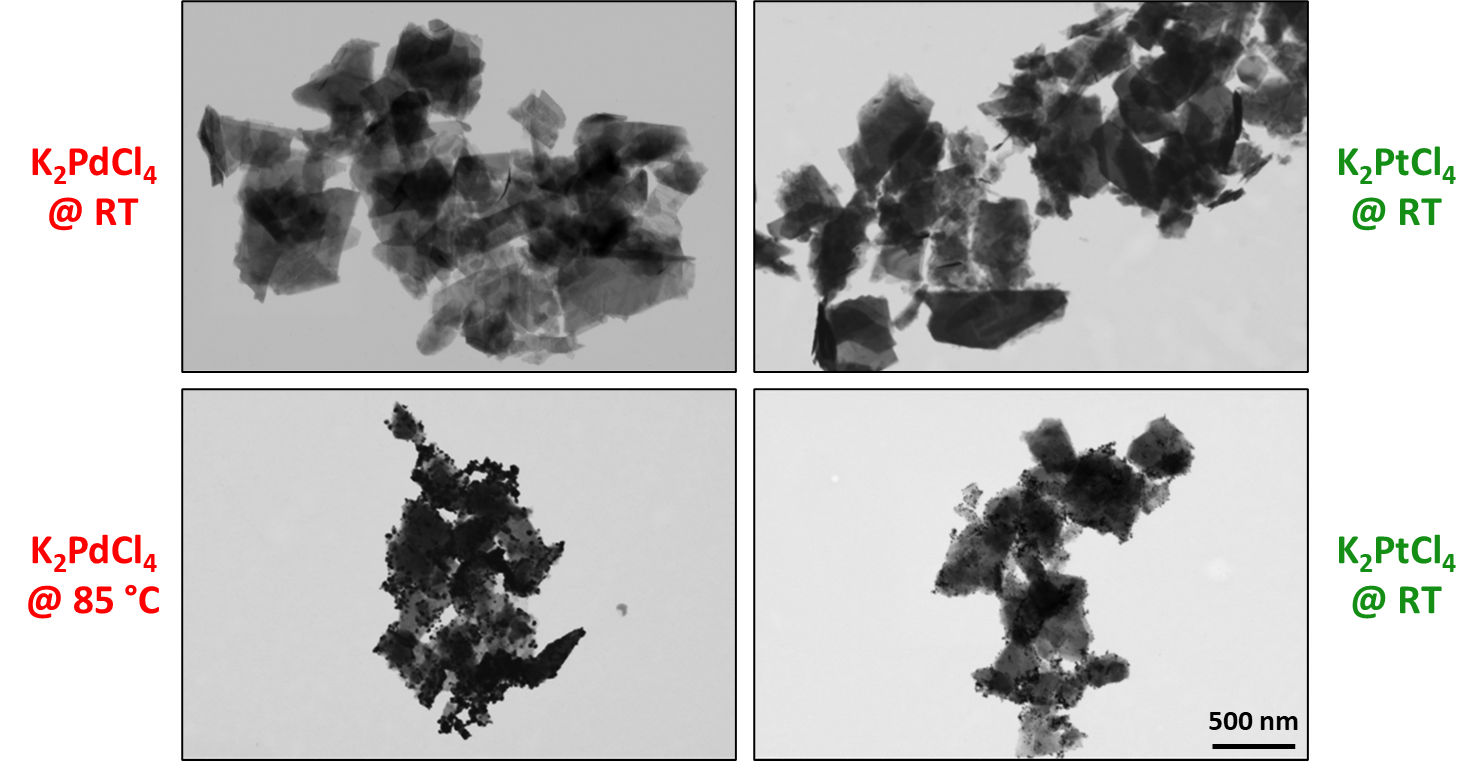


**Supplementary Fig. 3 | Energy-activated GD reaction.** Typical STEM images (same magnification) collected on MoS_2_ nanosheets upon reaction with K_2_PdCl_4_ and K_2_PtCl_4_ at room temperature (RT) and 85 °C. The syntheses are performed with one addition of a 125 mM stock aqueous solution of metal tetrachloride precursor to MoS_2_ dispersion.

We also evaluate the influence of temperature on the synthesis of edge-decorated TMDs with metal NPs. In fact, temperature plays a crucial role during the nucleation and growth processes of NPs, as it directly affects the adsorption and diffusion of the metal precursor on TMDs, the reaction kinetics and mechanisms. To this end, the reaction between MoS₂ and metal precursors of Pd and Pt (*i.e.*, K_2_PdCl_4_ and K_2_PtCl_4_, respectively) is explored at both room temperature (RT) and 85 ºC. In fact, the reactivity of such precursors towards TMDs (at RT) is lower due to a worst energy level alignment, unlike HAuCl_4_ whose reaction is already quite favorable at RT. The STEM images in Supplementary Fig. 3 demonstrate that the synthesis at 85 °C leads to a more favorable growth of Pd and PtNPs compared to RT, confirming the energy-activated nature of the GD mechanism.

1. **Galvanic displacement mechanism**


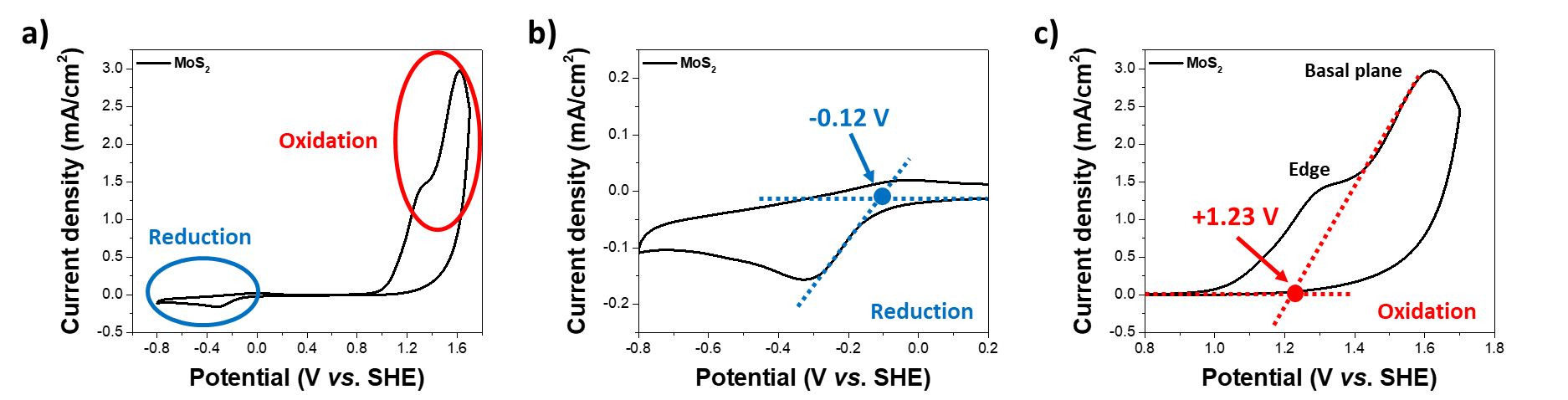


**Supplementary Fig. 4 | Cyclic voltammetry of solution-processed MoS_2_.** **a**, Cyclic voltammogram of solution-processed MoS_2_ (deposited by drop casting on a screen-printed carbon electrode), using scan rate equal to 50 mV/s and KCl 0.1 M_(aq)_ as supporting electrolyte. **b**, Magnification of the potential window from
-0.8 V to +0.2 V showing the MoS_2_ reduction peak, as well as the extrapolation of onset potential for the reduction reaction. **c**, Magnification of the potential window from +0.8 V to +1.7 V showing the typical MoS_2_ oxidation peak, as well as the extrapolation of onset potential for the oxidation reaction.

In order to prove the GD mechanism, based on a suitable energy level matching between the TMD electron affinity and redox potential of the metal precursors, we characterize all systems via cyclic voltammetry (CV). Supplementary Fig. 4 shows the CV for solution-processed MoS_2_, highlighting the typical reduction and oxidation peaks. It is worth noting that for the oxidation reaction we observe two distinct peaks, ascribed to edge (lower potential) and basal plane (higher potential) oxidation, confirming the higher reactivity of the former towards functionalization reactions^[1]^. Furthermore, by extrapolating the onset potential for the reduction and oxidation reaction (-0.12 V and +1.23 V for MoS_2_, respectively), we calculate the bandgap E_bandgap_ = +1.23 V – (-0.12 V) = +1.35 V = 1.35 eV, in agreement with data already reported in literature for few-layer MoS_2_^[2]^.


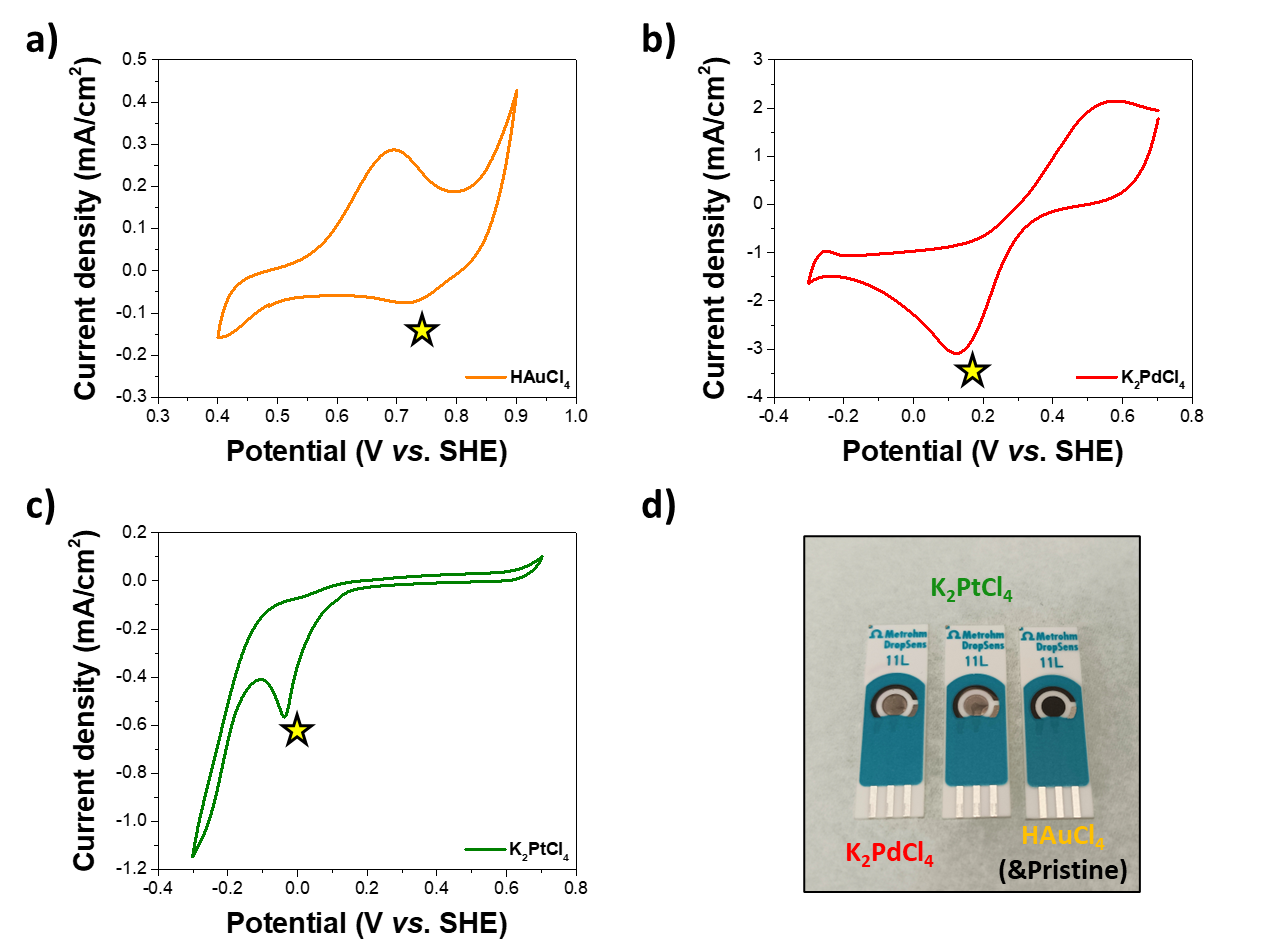


**Supplementary Fig. 5 | Cyclic voltammetry of metal tetrachloride complexes**. Cyclic voltammogram of
**a,** HAuCl_4_, **b**, K_2_PdCl_4_ and **c**, K_2_PtCl_4_ aqueous solutions with concentration equal to 25 mM for all complexes. No supporting electrolyte is used. All measurements are performed using screen-printed carbon electrodes and scan rate equal to 50 mV/s. The star indicates the reduction peak for each system. **d**, Optical image of spent screen-printed carbon electrodes after CV, showing the irreversible reduction reaction (under our operating conditions) for K_2_PdCl_4_ and K_2_PtCl_4_ as highlighted by the thin Pd(0) and Pt(0) layer deposited on the electrode after CV. Conversely, reversible reduction reaction is observed for HAuCl_4_, whose electrode remains pristine after CV.

Similarly, we perform CV on the noble metal precursors used for the GD reaction with TMDs, recording their reduction potentials. The data in Supplementary Fig. 5 reveals an irreversible reduction reaction for K_2_PdCl_4_ and K_2_PtCl_4_, in contrast to the behavior observed for HAuCl_4_. The reduction reactions involved, as well as the related experimental E_red_ values obtained, are the following:

$${[Au{Cl}_{4}]}^{-}+3e^{-}\leftrightarrow Au+4{Cl}^{-} E_{red}=+0.73 V$$

$${[Pd{Cl}_{4}]}^{2-}+2e^{-}\leftrightarrow Pd+4{Cl}^{-} E_{red}=+0.13 V$$

$${[Pt{Cl}_{4}]}^{2-}+2e^{-}\leftrightarrow Pt+4{Cl}^{-} E_{red}=-0.05 V$$

To corroborate the rection mechanism, in Supplementary Fig. 6 we report the energy diagram for MoS_2_ and metal precursors, as well as highlighting the driving force of GD.


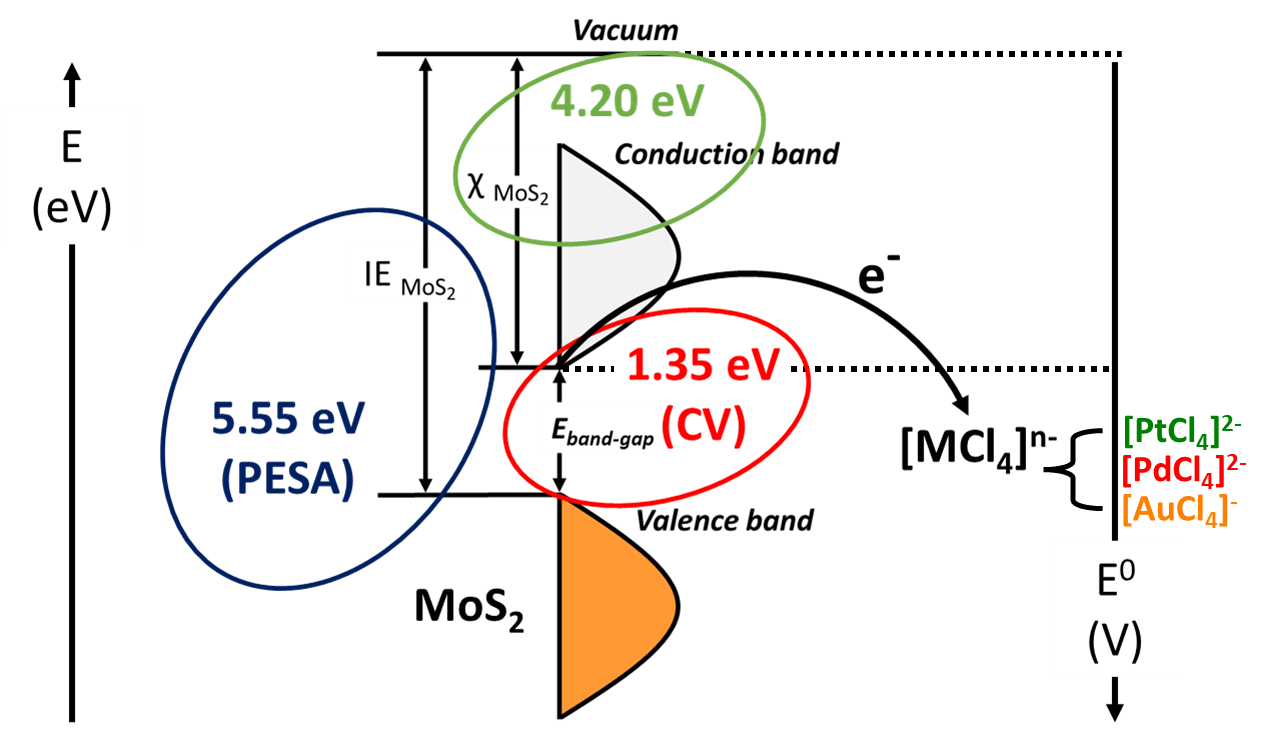


**Supplementary Fig. 6 | Galvanic displacement mechanism.** Energy diagram showing the position of the energy bands with respect to vacuum and electrochemical scale (*vs*. SHE) for the noble metal precursors and MoS_2_. About the latter, the sketch shows the measured ionization energy (IE) and bandgap (via PESA and CV, respectively), as well as the calculated energy affinity (χ), whose value governs the GD reaction.

As mentioned, the χ_MoS2_ governs the GD reaction. In fact, converting its value in electrochemical scale *(vs*. normal hydrogen electrode, NHE), we obtain E_red_ for MoS_2_ equal to -0.30 V, in good agreement with the value observed in the cyclic voltammogram reported in Supplementary Fig. 4b (reduction peak). The E_red_ for MoS_2_ is more negative than the values obtained for [MCl_4_]^n-^. Thus, considering the well-known electrochemistry rule for which the reagent with higher (more positive) E_red_ tends to be reduced, while the reagent with lower (more negative) E_red_ tends to be oxidized, it is evident why the GD reaction is favorable when reacting MoS_2_ with the noble metal tetrachloride complexes under exam.

At his point, it follows that the higher ΔE_red_, the higher the reactivity, as supported by the reaction of MoS_2_ with K_2_PdCl_4_ and K_2_PtCl_4_, which leads to NPs characterized by smaller size and lower loading compared to HAuCl_4_ and AuNPs (Supplementary Fig. 12). The same GD mechanism governs the reaction of WS₂ with the noble metal precursors, as highlighted by the CV results being analogous to those recorded for MoS₂ (Supplementary Fig. 7).


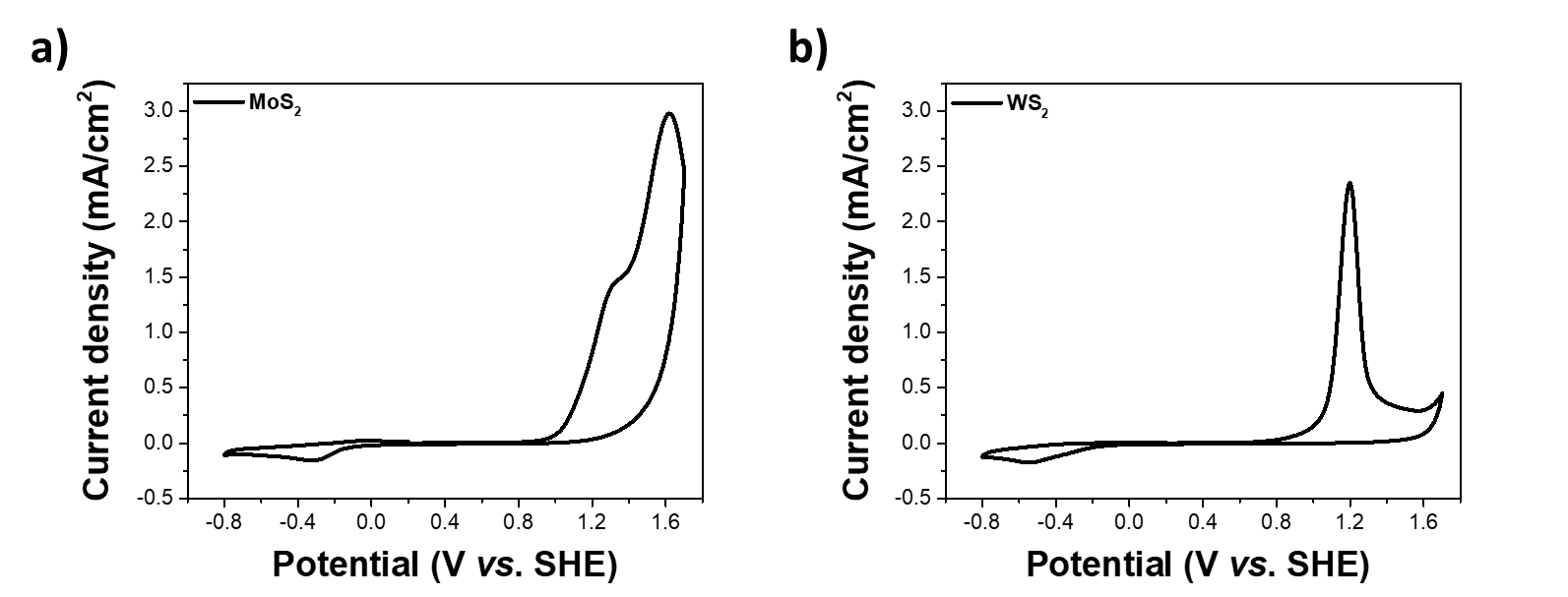


**Supplementary Fig. 7 | Cyclic voltammetry of MoS_2_ and WS_2_.** Cyclic voltammograms collected for solution-processed **a**, MoS_2_ and **b**, WS_2_ using scan rate equal to 50 mV/s and KCl 0.1 M_(aq)_ as supporting electrolyte.

1. **Density functional theory (DFT) calculations**

The interaction between [MCl_4_]^n-^ complexes and 2H-phase TMDs is analyzed based on the framework of spin-polarized density functional theory (DFT), within the PBE generalized gradient approximation (GGA) for the exchange-correlation functional and the PAW method^[3]^ using the Vienna ab initio simulation package (VASP)^[4]^. The wave functions are expanded in plane waves up to a kinetic energy cut-off of 400 eV. The Brillouin zone is sampled by 3x3x1 K-points using the Monkhorst–Pack scheme^[5]^. Periodic boundary conditions are applied for all calculations with a gap between the surfaces of 30 Å. The dispersion corrections are included through the standard D2 Grimme parameterization^[6]^. We investigate the GD reaction coordinate via DFT, starting from the adsorption of [MCl_4_]^n-^ metal precursors on the TMD surface to the formation of related metal NPs. To this end, we use HAuCl_4_ as a case study (Supplementary Fig. 8).


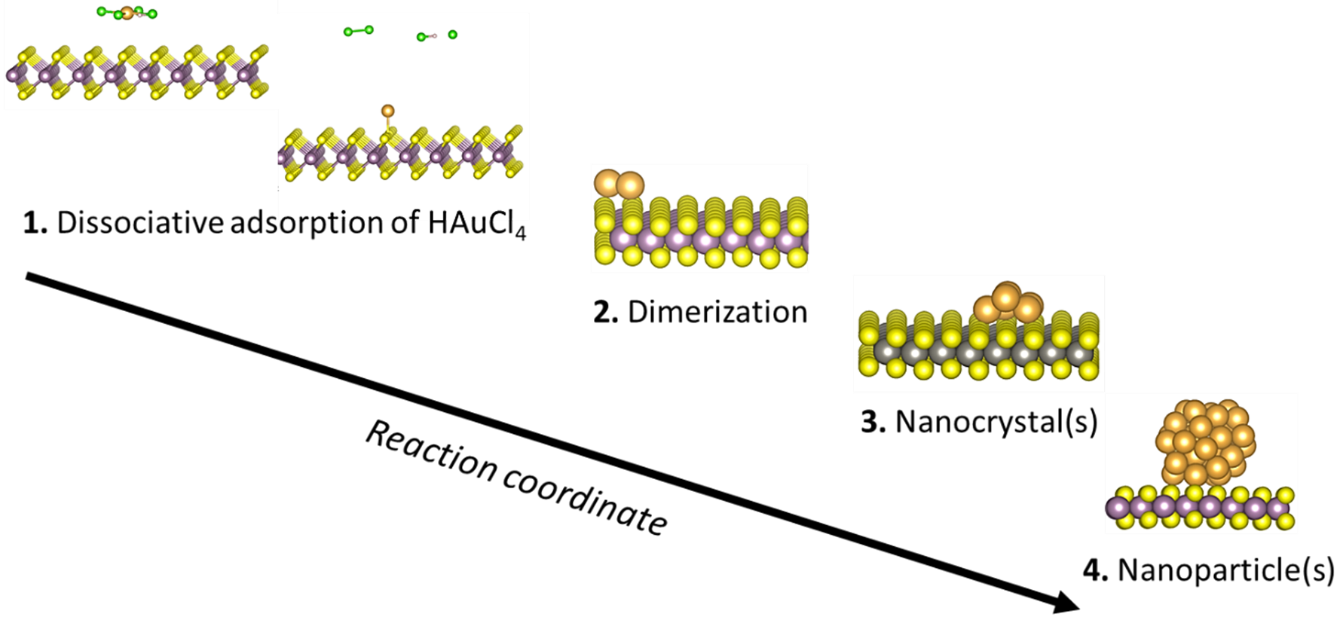


**Supplementary Fig. 8 | DFT calculations.** Sketch of the reaction coordinate between TMDs and HAuCl_4_, starting from the adsorption of the latter until the formation of AuNPs.

In particular, we first calculate the adsorption, dissociative and desorption energies for the MoS_2_/WS_2_ interacting with HAuCl_4_, considering pristine and defective 2D systems. We observe: (i) V_S_ favor the adsorption of metal precursors; (ii) the desorption probability of Cl-species is higher for MoS_2_ than WS_2_, due to the more negative surface charge of the former, leading to stronger repulsive interaction and, thus, faster desorption of Cl-species (Supplementary Fig. 9).


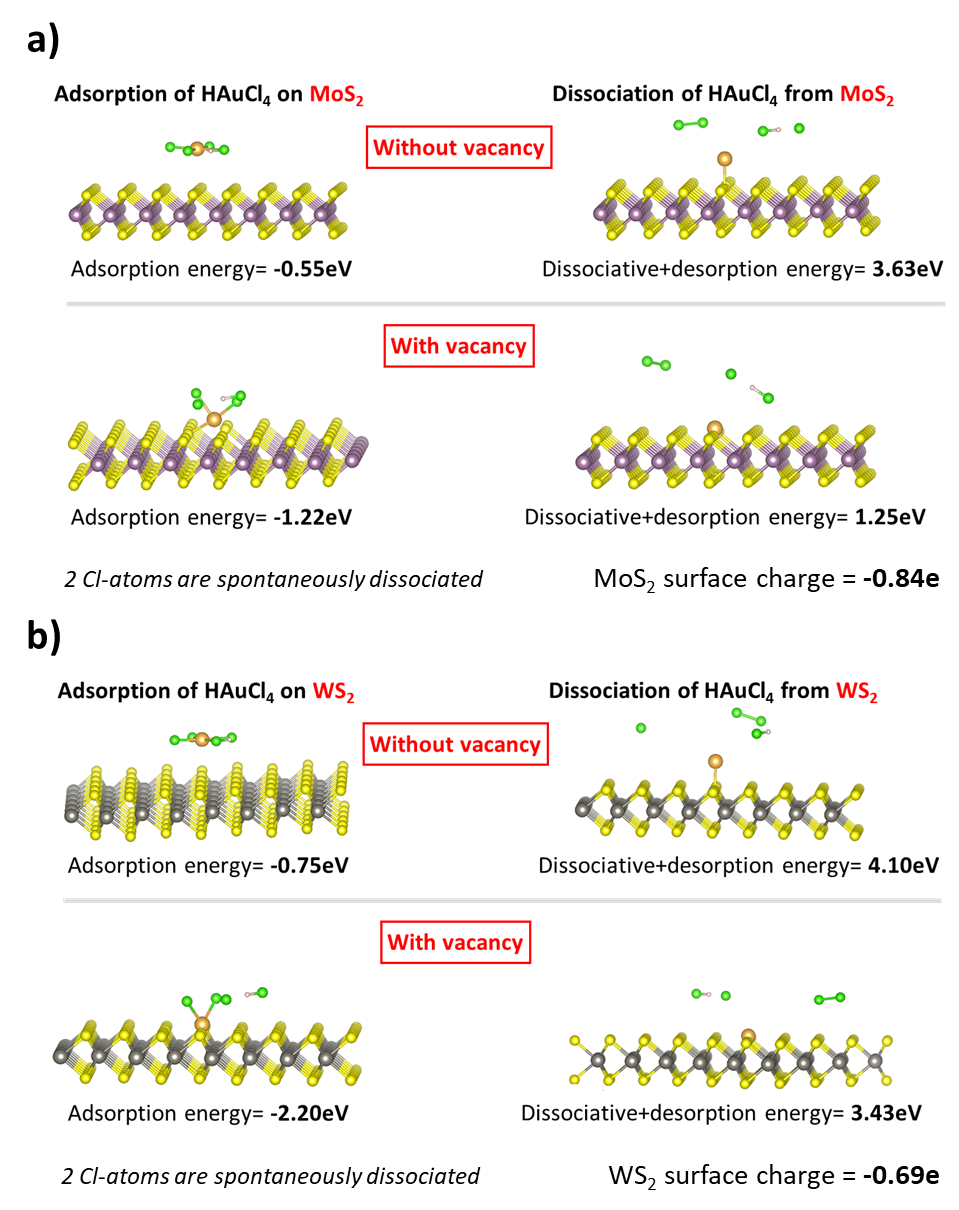


**Supplementary Fig. 9 | DFT calculations: MoS_2_/WS_2_ interacting with HAuCl_4_.** DFT-calculated adsorption, dissociative and desorption energy for **a**, MoS_2_ and **b**, WS_2_ considering pristine and defective 2D crystals. Moreover, because of the more negative surface charge of MoS_2_ (-0.84e) compared to WS_2_ (-0.69e), the metal precursor adsorption and the formation of initial metal nuclei result more favorable.


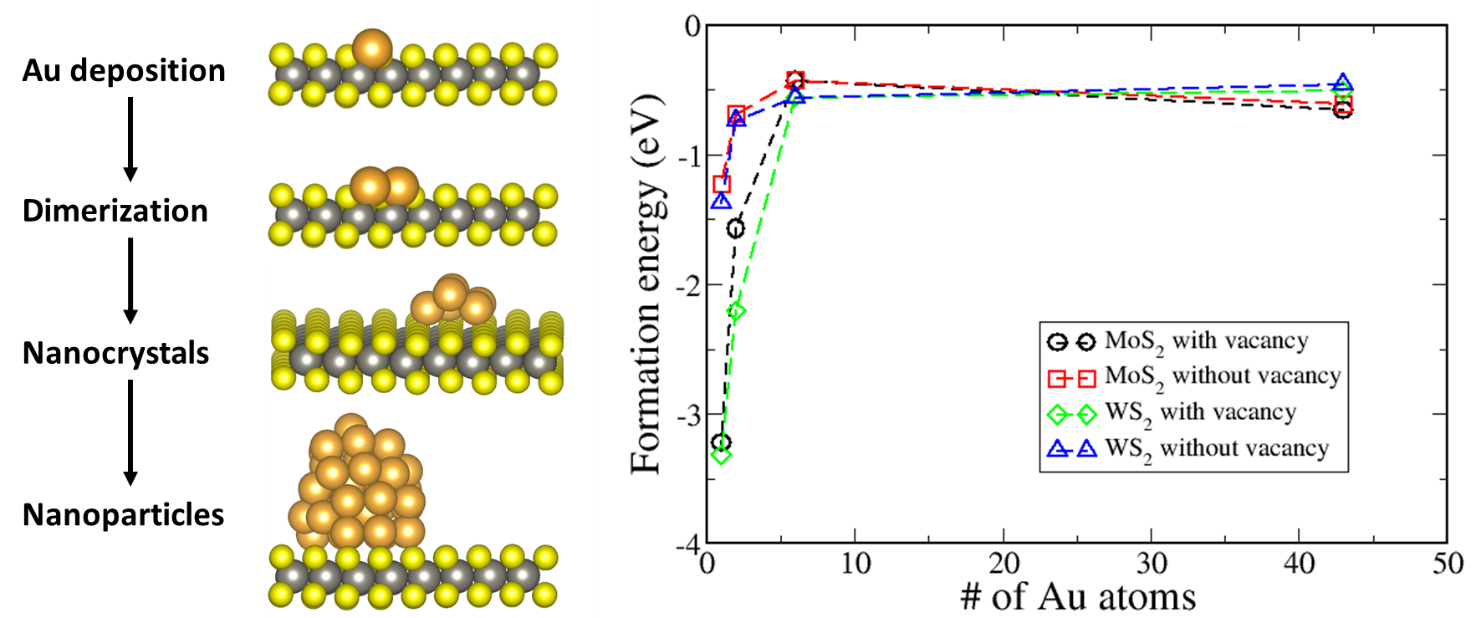


**Supplementary Fig. 10 | DFT calculations: energy formation.** DFT calculations about the formation energy of Au clusters on MoS_2_/WS_2_ crystals with and without V_S_. The presence of V_S_ makes the nucleation and growth process much more favorable (*viz.*, more negative formation energy) during the initial steps of the GD reaction.

We also confirm the mechanistic features of the GD reaction via DFT calculations. In fact, from the MoS_2_ Bader analysis reported below, we observe two electron-transfer processes taking place (i) during the metal precursor adsorption (electron transfer from MoS_2_ to HAuCl_4_) and (ii) upon AuNP formation (electron transfer from AuNPs to MoS_2_).


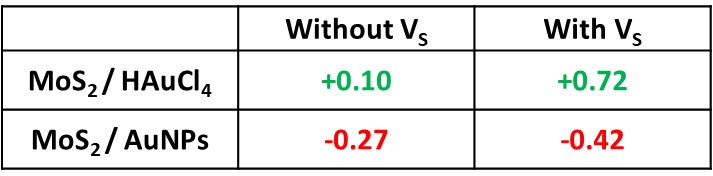


**Supplementary Table 1 | MoS_2_ Bader analysis.** Bader analysis for GD reaction between MoS_2_ and HAuCl_4_, showing two distinct electron transfer processes. Positive sign (green): MoS_2_ loses electrons; Negative sign (red): MoS_2_ gains electrons.

Finally, Bader analysis also confirms the crucial role played by V_S_ in making the overall reaction more favorable, considering the stronger electron transfer calculated for this case in Supplementary Table 1 (*viz.*, more positive and negative values for the two electron-transfer steps).

1. **Multiscale characterization of edge-decorated TMDs with metal NPs**


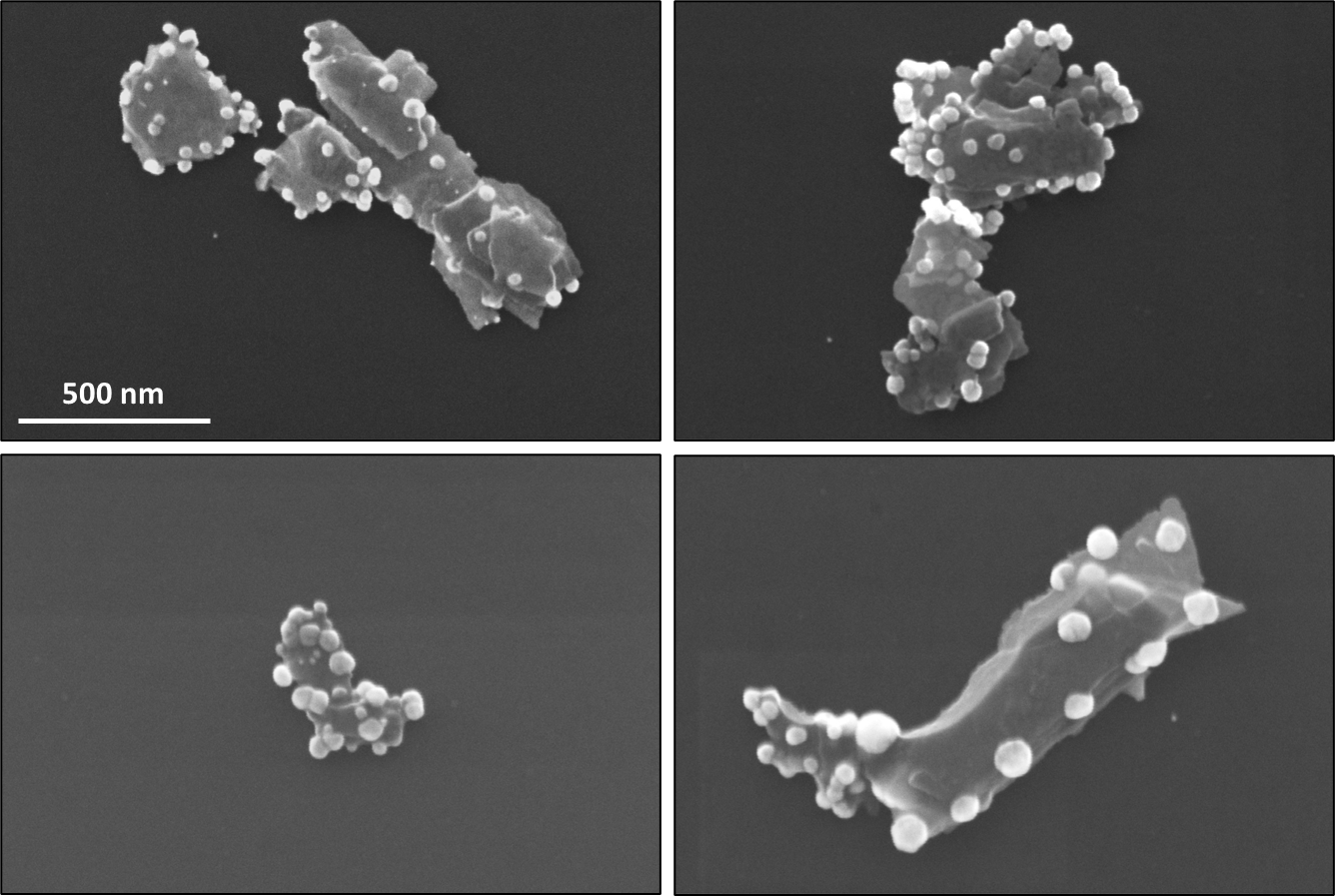


**Supplementary Fig. 11 | TMDs + metal NPs via GD mechanism.** Typical SEM images of selectively edge-decorated MoS_2_ + AuNPs nanosheets obtained via GD reaction.


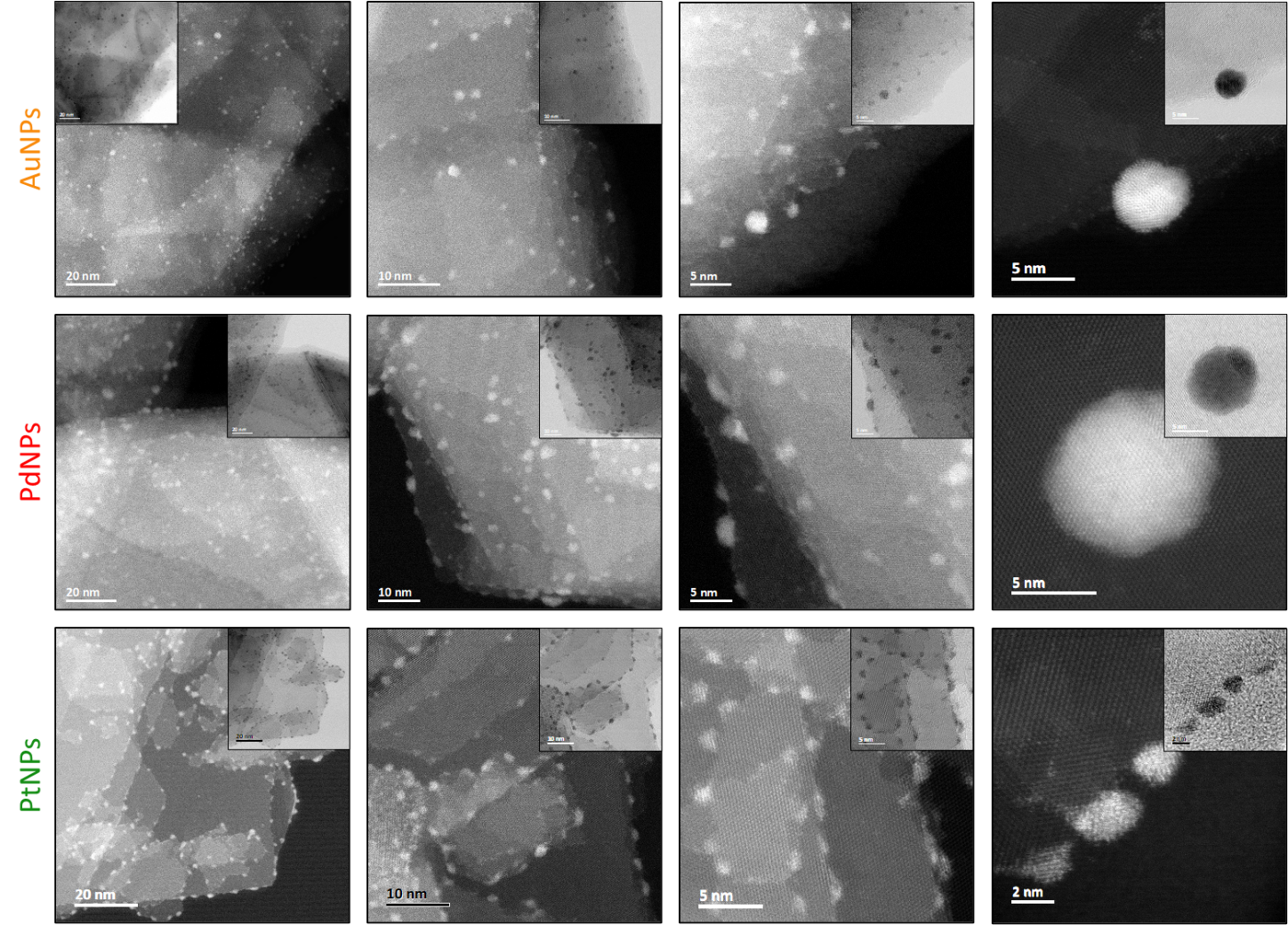


**Supplementary Fig. 12 | Characterization of edge-decorated TMDs + metal NPs.** Typical DF-HR-STEM images (Inset: BF) of edge-decorated MoS_2_ + AuNP, PdNPs, and PtNPs with increasing magnification from left to right. The synthesis steps are performed at room temperature using aqueous solutions of metal tetrachloride precursors.

To further investigate the morphological properties of metal NPs, we record HR-STEM images of MoS_2_ + AuNPs, PdNPs, and PtNPs. In agreement with the proposed GD growth mechanism, the metal NPs are predominantly localized along the edges of MoS_2_ nanosheets, which serve as the most defective and reactive sites, favoring the functionalization reaction.


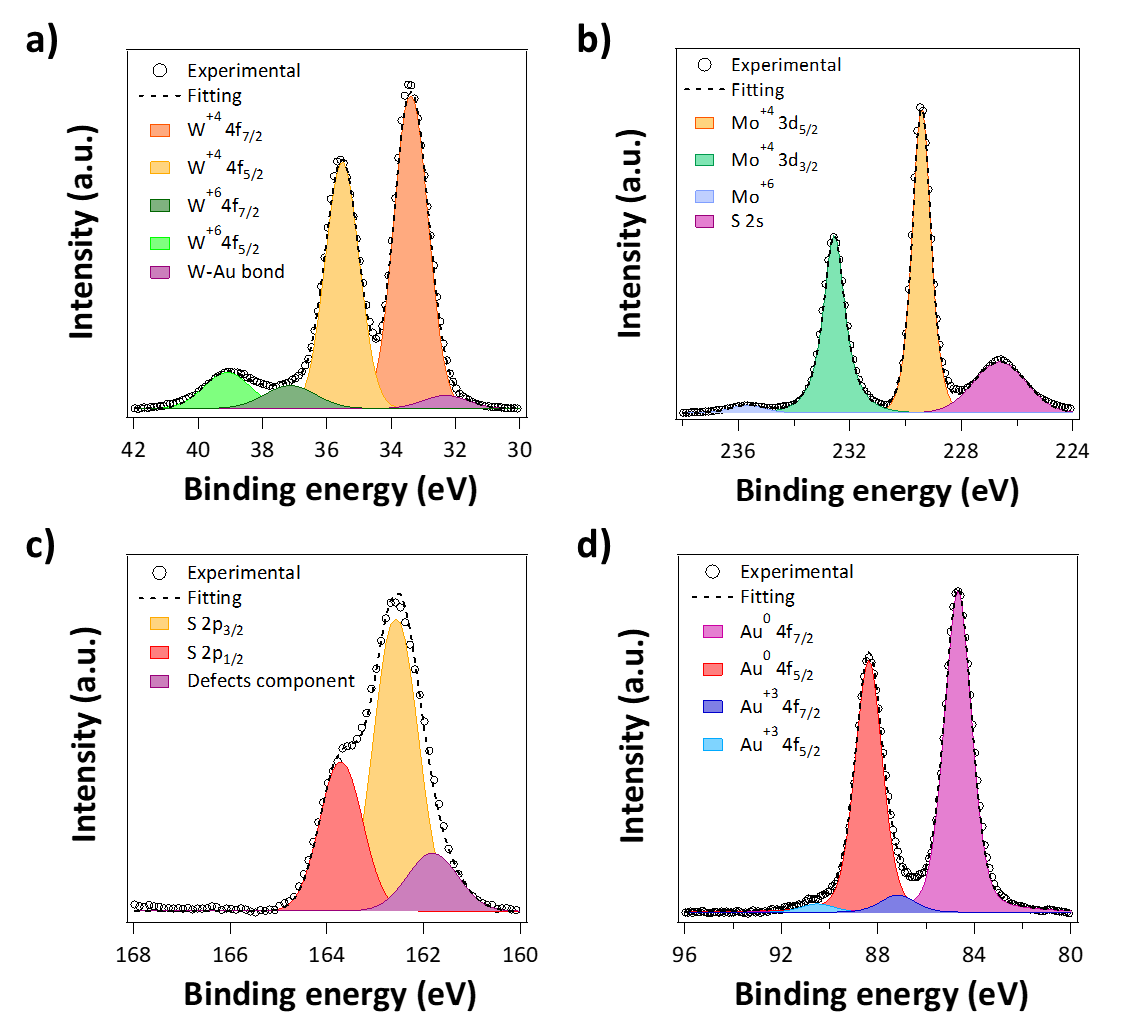


**Supplementary Fig. 13 | XPS of MoS_2_/WS_2_ + AuNPs.** High-resolution **a**, W 4f, **b**, Mo 3d, **c**, S 2p, and **d**, Au 4f XPS spectra for MoS_2_/WS_2_ + AuNPs.

We perform XPS analysis to confirm the absence of oxidation for MoS_2_ and WS_2_ upon GD reaction. Supplementary Fig. 13 displays the typical spectra for W 4f, Mo 3d, S 2p and Au 4f, where the presence of W^+6^ and Mo^+6^ is negligible and likely ascribed to minimal oxidation of 2D crystals during the exfoliation steps^[7]^. These results are in agreement with GIXRD patterns (Fig. 3), which show no signs of MoO_x_ species as a result of GD mechanism.

1. **Applications of edge-decorated TMDs with metal NPs**

Photothermal sensing

To assess the performance of hybrid systems made of MoS_2_ + metal NPs for photothermal sensing, we calculate the photothermal conversion efficiency η (PTCE) for each system under investigation using the following equation^[8]^:

$$\eta=\frac{hS\Delta T_{max}-Q_{S}}{P_{laser} (1-{10}^{-A_{785}})}$$

where P_laser_ indicates the laser power, A the absorbance of the materials at 785 nm (A_785_ ≈ 3 for all systems), Q_S_ the heat loss/dissipated from the cuvette. Since the cuvette is insulated with Styrofoam during the analysis, we consider Q_S_ = 0. The parameters needed to calculate the PTCE can be obtained through the following equations:

$$\vartheta=\frac{T-T_{0}}{T_{max}-T_{0}}$$

where T_0_ represents the environmental temperature and T_max_ the maximum temperature reached by each system during the analysis.

$$ln\vartheta=-\frac{t}{\tau_{S}}$$

with τ_S_ the characteristic cooling time extrapolated from the slope of “lnθ *vs*. Time (t)” plot.

$$hS=\frac{m_{D}C_{H_{2}O}}{\tau_{S}}$$

where m_D_ represent the mass of the dispersions, C_H2O_ the specific heat of water, h the heat transfer coefficient, and S the cuvette surface area.


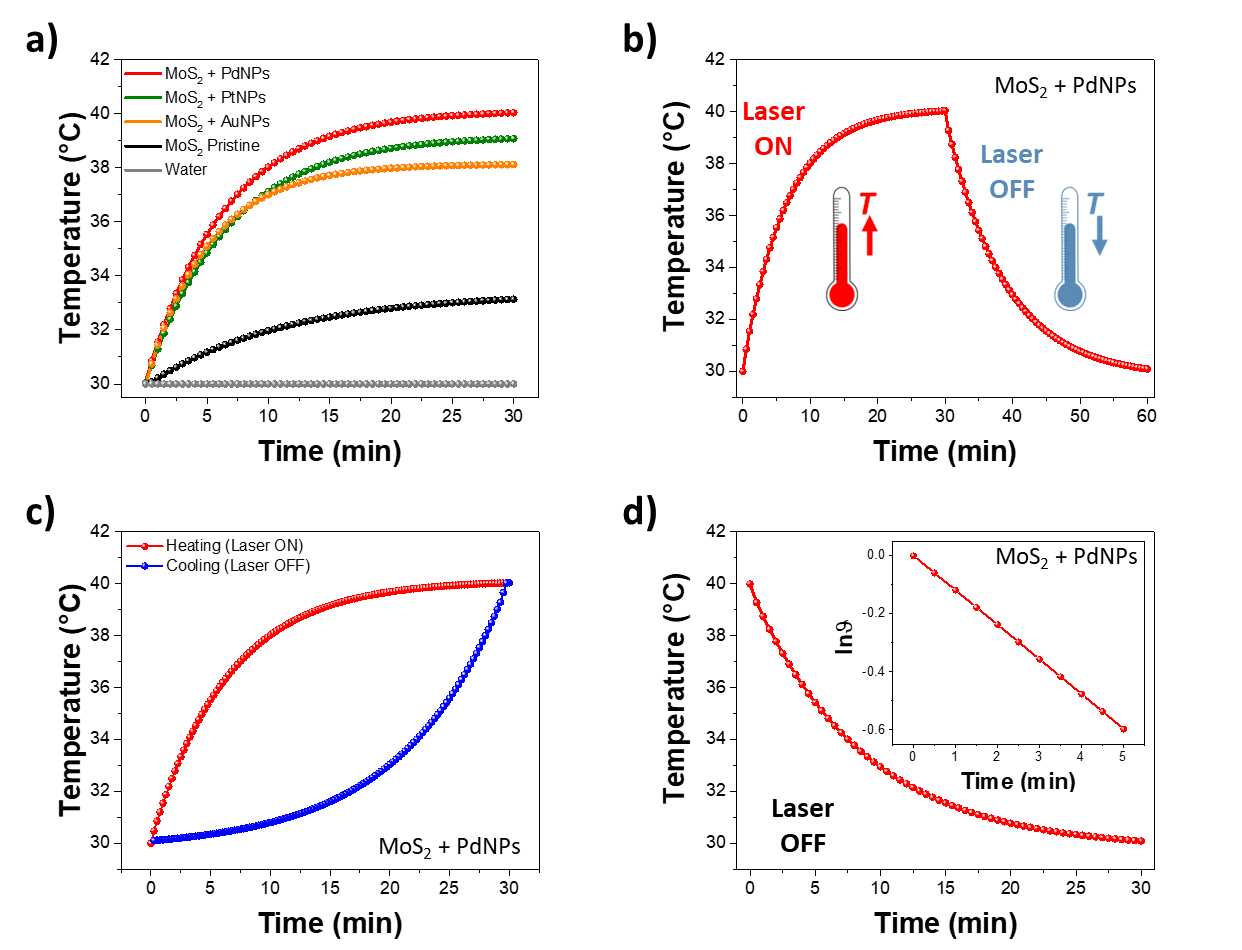


**Supplementary Fig. 14 | Photothermal sensing.** **a**, Photothermal heating curves for MoS_2_ + metal NPs (400 µg/mL). **b**, Photothermal heating and cooling curve for MoS_2_ + PdNPs (400 µg/mL) and **c**, related hysteresis loop. **d**, Cooling curve for MoS_2_ + PdNPs (400 µg/mL). *Inset:* lnθ *vs*. Time plot for MoS_2_ + PdNPs (400 µg/mL) from which it is possible to extrapolate (from its slope) the characteristic cooling time and calculate the PTCE.


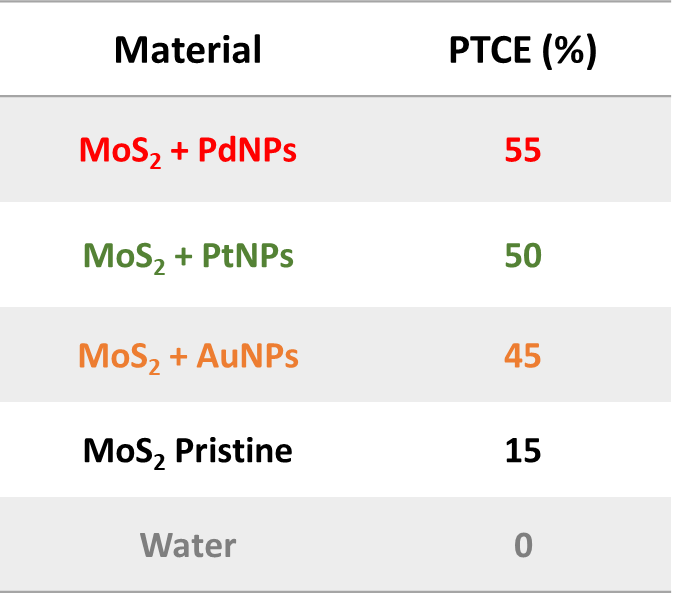


**Supplementary Table 2 | Photothermal Conversion Efficiency.** PTCE for MoS_2_ + metal NPs (at 785 nm), showing MoS_2_ pristine and water for comparison.

Optical sensing – SERRS

In our study, we use plasmonic platforms instead of colloidal NPs in solution for SERRS due to their superior stability, reproducibility, and performance. Their stable nature and ease of handling also make them better suited for practical applications, such as biosensing and environmental monitoring. The plasmonic substrates are prepared by filtering MoS₂ + AuNPs aqueous dispersions and dry transferring them onto an ultrathin PET film using a simple pressing technique (Methods). This process ensures a stable and functional platform for reliable SERRS measurements. We optimize the loading of AuNPs on MoS_2_ nanosheets in order to prepare ideal SERRS platforms. To this end, we produce three batches with increasing loading (referred to as low, medium and high) and evaluate their SERRS performance (Methods and Supplementary Fig. 15).


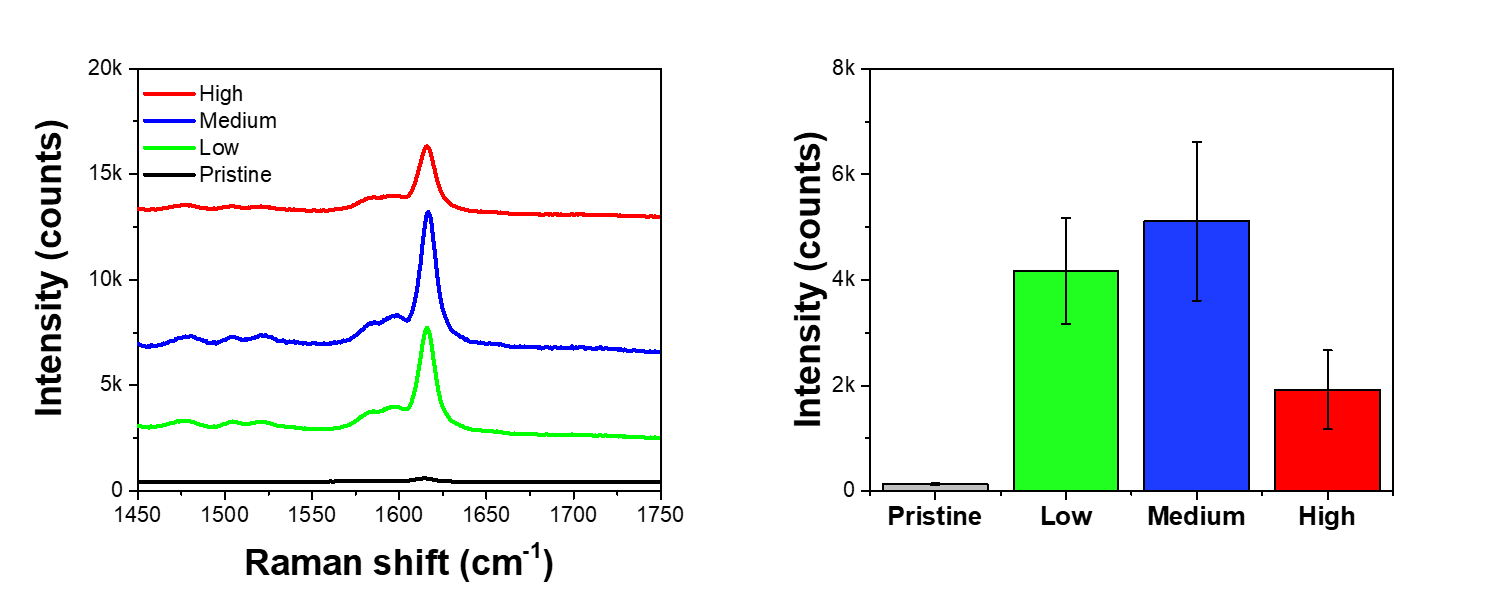


**Supplementary Fig. 15 | Optical sensing**. Comparison of average SERRS intensity collected for MoS_2_ + AuNPs samples with different loadings, using MGITC as analyte.

We observe an enhanced (average) intensity when moving from low to medium loading, because of higher SERRS signals caused by the coupling of AuNPs. However, high-loading sample shows lower average intensity compared to medium loading, due to an excessive amount of NPs on MoS_2_ flakes which hinders their coupling (because of quantum effects) and, therefore, the SERRS performance^[9]^. For these reasons, MoS_2_ + AuNPs sample with medium loading is used for the optical sensing analysis reported in the Main Text (Fig. 4).

Electrocatalysis

To further explore the versatility of applications and validate the performance of MoS_2_ + metal NPs for electrocatalysis, we perform electrochemical impedance spectroscopy (EIS), using the same experimental setup employed for CV investigations, as well as keeping constant the sample amount and size of NPs. By using as equivalent circuit a second-order Voigt circuit, we extrapolate the charge transfer resistance (R_CT_) for all systems, which represents a key parameter to assess the performance of such materials for electrocatalytic applications^[10]^. The results confirm the activity trend reported in the Main text, with the ascending order of R_CT_ as follows: PtNPs (≈ 10 Ω)< PdNPs (≈ 25 Ω) < AuNPs (≈ 70 Ω) < Pristine (≈ 410 Ω). Our findings highlight the superior catalytic activity of PtNPs for HERs and demonstrate the overall enhanced performance of MoS_2_ + metal NP systems compared to pristine MoS_2_.


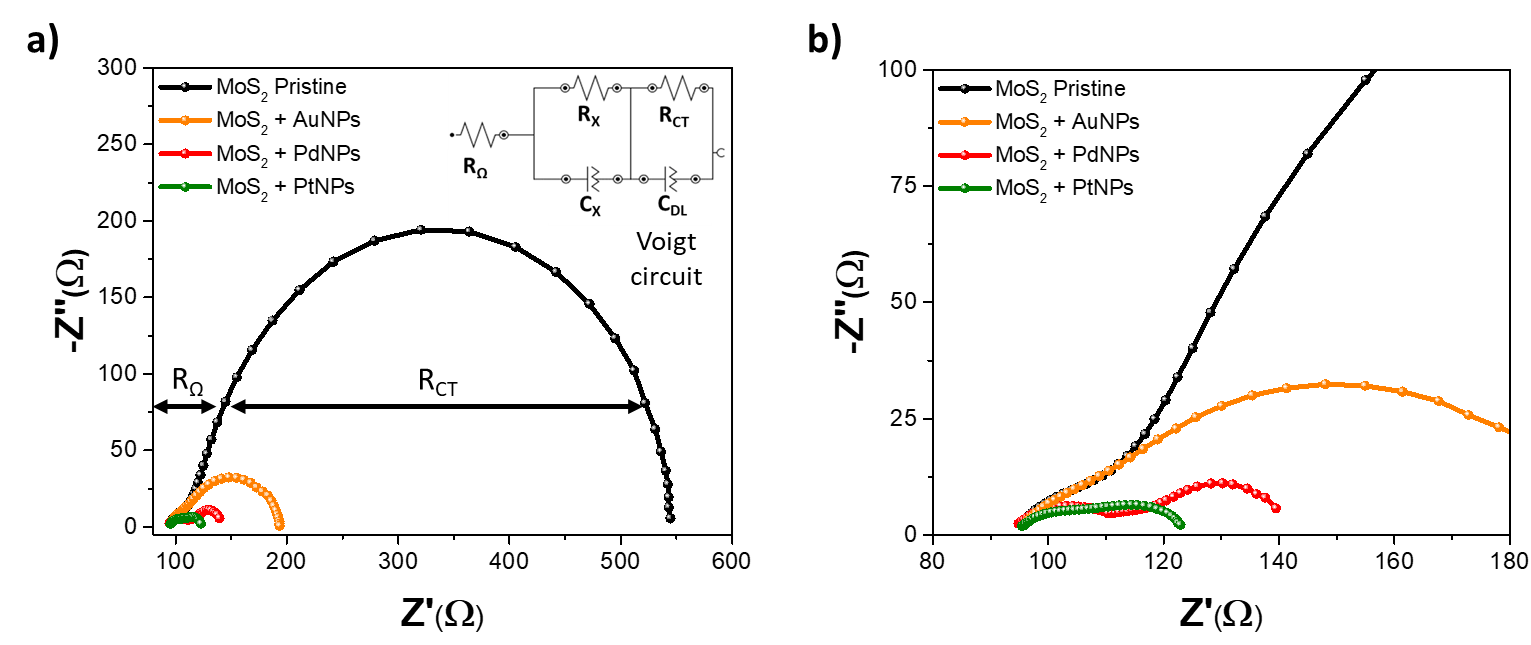


**Supplementary Fig. 16 | Electrocatalysis.** **a**, Nyquist plots for MoS_2_ + metal NPs and **b**, related magnification plot to better visualize R_CT_ for all systems. The EIS experiments are performed by using the same experimental setup employed for CV analysis, with V_DC_ = -1 V. The acidic electrolyte used is 0.5 M H_2_SO_4_.

Electronics

To further explore the performance of MoS_2_ + AuNPs in electronic devices, we record their operation stability over intense cycling (250 cycles) and compare their operating current (I_DS_) to the leakage current (I_GS_), with the latter being several orders of magnitude lower (Supplementary Fig. 17).


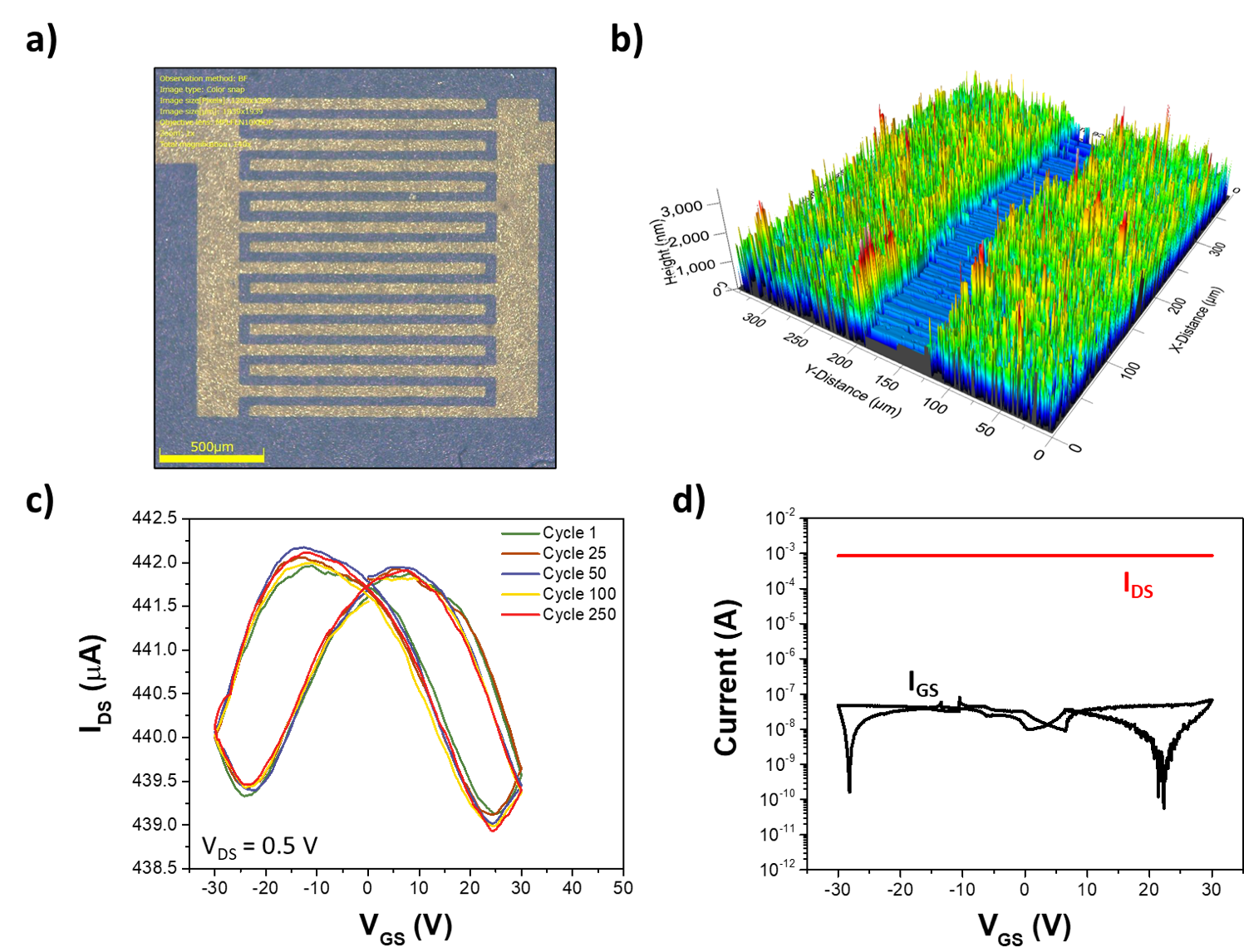


**Supplementary Fig. 17 | Electronics.** **a**, Optical image of a MoS_2_ + AuNPs electronic device and related **b**, optical profilometry performed to assess the film thickness (ca. 400 nm). **c**, Transfer curves recorded over 250 cycles for MoS_2_ + AuNPs (high loading) device (scan rate ≈ 10 V/s). The “butterfly” curve was recorded for all five MoS_2_ + AuNPs (high loading) devices tested. **d**, Transfer curve (semi-log scale) for MoS_2_ + AuNPs (high loading) device (I_DS_ *vs*. V_GS_) and its leakage current (I_GS_ *vs*. V_GS_), with V_DS_ = 0.5 V.

1. **Captions for the supplementary videos**

Supplementary Video 1: In-situ liquid STEM experiment showing the toposelective growth of AuNPs (using HAuCl_4_ as precursor) at the edges of solution-processed MoS_2_ flakes via GD.

Supplementary Video 2: Electrocatalytic activity of MoS_2_ + PtNPs material (deposited on a screen-printed electrode) during hydrogen evolution reaction experiment. The potential is swept from 0 V to -0.8 V, using 0.5 M H_2_SO_4_ as acidic electrolyte.

Additional information can be found in the “Methods” section of the manuscript.

1. **References**

[1] A. Y. S. Eng, A. Ambrosi, Z. Sofer, P. Šimek, M. Pumera, *ACS Nano* **2014**, 8, 12185.

[2] K. F. Mak, C. Lee, J. Hone, J. Shan, T. F. Heinz, *Phys. Rev. Lett.* **2010**, 105, 136805.

[3] J. P. Perdew, K. Burke, M. Ernzerhof, *Phys. Rev. Lett.* **1996**, 77, 3865.

[4] G. Kresse, J. Furthmüller, *Phys. Rev. B* **1996**, 54, 11169.

[5] H. J. Monkhorst, J. D. Pack, *Phys. Rev. B* **1976**, 13, 5188.

[6] S. Grimme, *J. Comput. Chem.* **2006**, 27, 1787.

[7] S. Ippolito, A. G. Kelly, R. Furlan de Oliveira, M.-A. Stoeckel, D. Iglesias, A. Roy, C. Downing, Z. Bian, L. Lombardi, Y. A. Samad, V. Nicolosi, A. C. Ferrari, J. N. Coleman, P. Samorì, *Nat. Nanotechnol.* **2021**, 16, 592.

[8] C. M. Hessel, V. P. Pattani, M. Rasch, M. G. Panthani, B. Koo, J. W. Tunnell, B. A. Korgel, *Nano Lett.* **2011**, 11, 2560.

[9] A. B. Serrano-Montes, J. Langer, M. Henriksen-Lacey, D. Jimenez de Aberasturi, D. M. Solís, J. M. Taboada, F. Obelleiro, K. Sentosun, S. Bals, A. Bekdemir, F. Stellacci, L. M. Liz-Marzán, *J. Phys. Chem. C* **2016**, 120, 20860.

[10] H. Li, K. Yu, C. Li, Z. Tang, B. Guo, X. Lei, H. Fu, Z. Zhu, *Sci. Rep.* **2015**, 5, 18730.
